# Effect of metal substitution on polymorphism and flexibility of 1D coordination polymers

**DOI:** 10.1039/d6ce00723f

**Published:** 2026-11-05

**Authors:** Kyriaki Koupepidou, Asif Raza, Tao He, Alan C. Eaby, Soumya Mukherjee, Michael J. Zaworotko

**Affiliations:** a Bernal Institute, Department of Chemical Sciences, University of Limerick Limerick V94T9PX Republic of Ireland; b Institute for Integrated Cell-Material Sciences, Kyoto University Institute for Advanced Study, Kyoto University Yoshida Ushinomiya-cho, Sakyo-ku Kyoto 606-8501 Japan koupepidou.kyriaki.2r@kyoto-u.ac.jp

## Abstract

Coordination polymers (CPs) can exhibit significant changes in properties when they adopt different polymorphs. Here, we show that metal substitution can alter the polymorphic outcome and framework stability of a one-dimensional (1D) CP. We report two packing polymorphs of a Cu(ii)-based CP, constructed from 1,3-bis(1*H*-imidazol-1-yl)benzene (bib, 1), namely polymorphs A and B of {[Cu(bib)_2_(H_2_O)][SiF_6_]}_*n*_, **[SP-1-Cu-H_2_O][SIFSIX]-A** and **[SP-1-Cu-H_2_O][SIFSIX]-B**, wherein extra-framework SiF_6_^2−^ (**SIFSIX**) anions are hydrogen-bonded to coordinated aqua ligands (H_2_O). The polymorphs comprise spiro (**SP**) loops arising from the bent conformation of the linker ligand (bib), but differ in the packing of their 1D chains. The methanol (MeOH) precursor of polymorph **A**, {[Cu(bib)_2_(CH_3_OH)_2_][SiF_6_]}_*n*_, **[SP-1-Cu-2MeOH][SIFSIX]-A**, crystallised concomitantly with **[SP-1-Cu-H_2_O][SIFSIX]-B** and converted to **[SP-1-Cu-H_2_O][SIFSIX]-A** on exposure to air, through a single-crystal-to-single-crystal replacement of methanol by water. Metal substitution from Cu(ii) to Zn(ii) afforded only one crystalline phase, **[SP-1-Zn-2MeOH][SIFSIX]-A**, which underwent a phase transformation to **[SP-1-Zn-2H_2_O][SIFSIX]-A** upon heating at 373 K under vacuum and subsequent exposure to air. Although the **A** polymorph of Cu(ii) was not stable to activation, the Zn(ii) analogue was successfully activated and analysed by gas (CO_2_ at 195 and 298 K) and vapour (water at 300 K) sorption. The insights presented herein demonstrate how metal substitution may affect polymorphic landscapes and alter the activation behaviour of 1D CPs.

## Abstract 2

Coordination polymers can exhibit significant changes in properties when they adopt different polymorphs.

## Graphical abstract

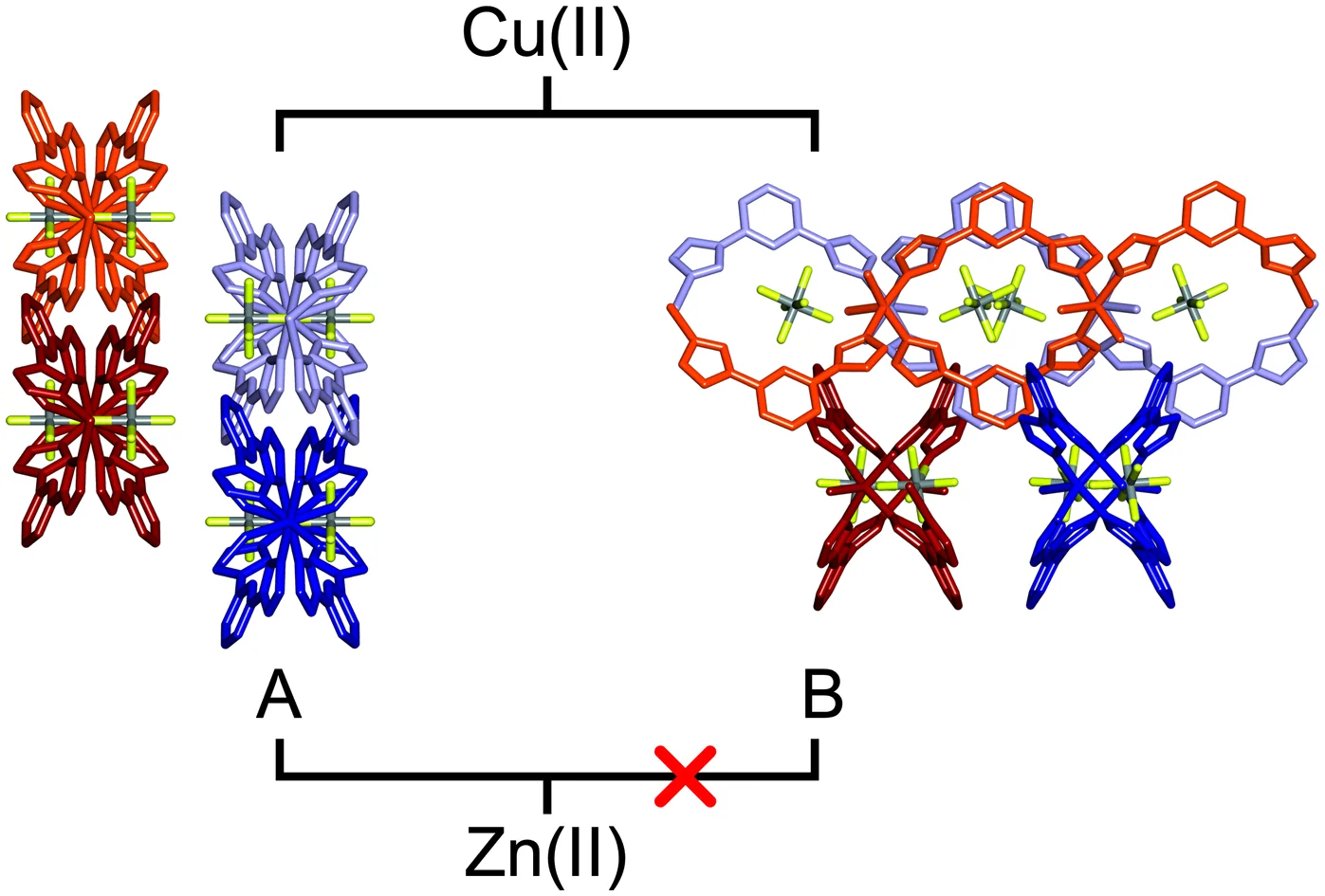

## Introduction

Coordination polymers (CPs),^1^ comprising inorganic nodes connected by organic linker ligands, are inherently modular, and therefore allow for chemical^2^ and topological^3^ diversity in the solid state. Owing to the multitude of connectivity modes of their molecular building blocks in three-dimensional space, the idea of building more than one type of network with the same chemical composition is realised in the concept of supramolecular isomerism, especially for flexible linker ligands.^4–6^ As a type of supramolecular isomerism, packing polymorphism is the existence of different crystal packings of the same chemical compound and is relevant in CPs, as exemplified by different network arrangements and pore structures.^7–9^ That pores often exist in CPs and can thereby accommodate guest molecules means that true CP polymorphs only exist in the absence of guests.^10^ The term pseudopolymorph was coined by pharmaceutical scientists to differentiate anhydrate molecular solids from their solvates and/or hydrates,^11,12^ and has also been used in the context of CPs.^13^ While pseudopolymorphs resulting from topological diversity are commonly encountered in CPs,^6,14–16^ packing or conformational polymorphism and the resulting implications on properties are seldom the focus of studies on CPs.

The importance of polymorphism in CPs rests on the idea that even small changes in crystal structure can impact physicochemical properties.^17^ This is especially so in the context of gas adsorption, where fine-tuning of pore size, shape, and chemistry (pore engineering) often affords significant changes in affinity to guest molecules,^18^ while changes in crystal packing can afford different CP adsorbents altogether. For example, the interpenetrated coordination network **SIFSIX-2-Cu-i** exhibits substantially higher CO_2_ uptakes under ambient conditions compared to its non-interpenetrated less dense polymorph, **SIFSIX-2-Cu**, despite its much lower surface area.^19^ Similar behaviour was observed in the recently discovered polymorph of **CALF-20**,^20,21^ with higher projected adsorption enthalpy for CO_2_ capture.^22^ Further, marked differences in adsorption profiles have been observed among packing polymorphs,^9^ and among polymorphs of different dimensionality.^23^ It is therefore appropriate to aim to control polymorphism in order to achieve desired properties. Generally, CP polymorphism, including interpenetration,^24^ can be realised through variation of temperature^25–28^ and pressure,^29^ or templated synthesis.^30,31^ To selectively access a particular polymorph, crystal engineering strategies^32^ such as metal substitution^33^ may be employed, while the choice of metal can also independently influence the resulting material properties. Nevertheless, control over and study of polymorphism can be exacerbated as activation and testing conditions often preclude the use of metastable polymorphs as adsorbents.^10,22^

CPs are widely studied for their porosity, owing to their tunable pore size and chemistry.^34^ It is possible to classify porosity in CPs as intrinsic, *i.e.*, porosity within the framework architecture, or extrinsic, *i.e.*, porosity that results from inefficient packing of networks.^35–37^ Two- or three- dimensional (2D, 3D) CPs (*i.e.*, coordination networks^38^) are most often evaluated for gas sorption and separation,^39^ despite one-dimensional (1D) CPs being ubiquitous in the literature.^40^ This may be due to the typical lack of intrinsic porosity in 1D CPs, although extrinsic porosity is possible when sustained through a combination of directional noncovalent interactions.^37^ 1D CPs can adopt a wide range of structural classes, including linear, zigzag, ladder, or ribbon motifs.^40^ Whereas all these motifs have the potential to form extrinsically porous structures, if the crystal packing is inefficient, ladder- and ribbon-type 1D CPs can also show intrinsic porosity.^41,42^ Further, spiro-linked (ribbon) CPs can be designed through the use of angular linkers that create a loop comprising two metal nodes and two linkers.^9,43–46^ These “spiro loops” can enable CPs with intrinsic porosity of narrow pore size, leading to ultramicroporous CPs (pore size <7 Å)^19^ which are recognised for their ability to exhibit tight adsorbate binding, a key requirement for effective gas separations.^9,44^

In addition to porosity, stability of CPs to heat and vacuum is critical for their evaluation in gas sorption, which requires the removal of solvent molecules present in the pores upon crystallization. In general, 1D CPs tend to be less thermally stable than 2D and 3D CPs.^47^ This may be attributed to the absence of coordination bonds along the two other directions, replaced by generally weaker noncovalent interactions, such as hydrogen bonding and dipole–dipole interactions.^48,49^ On the other hand, these weaker interactions are prone to rearrangement and/or breakage, providing 1D CPs with greater ease in undergoing structural transformations as a response to external stimuli.^47^ This phenomenon, known as flexibility,^50–52^ occurs only in a small subset of CPs.^53,54^ Kitagawa's classification of CPs into three different generations based on their ability to structurally respond to the presence or absence of guests^55^ has prompted significant advances in flexible CPs over the past two decades.^56,57^ Some 1D CPs show flexibility, including reversible transformations to higher dimensionality CPs^58^ or transformations from ladder to linear CPs,^59^ as well as phase transformations triggered removal or addition of solvent molecules.^60–62^ Nevertheless, such flexibility is scarcely reported for 1D CPs.

Spiro-linked CPs can exhibit flexibility arising from the angularity of the framework backbone.^44,63^ For example, bent dipyridyl linkers were used to obtain some of the earliest examples of flexible 1D CPs, including [Cu(bpe)_2_(PF_6_)_2_]_*n*_ (bpe = 1,2-bis(4-pyridyl)ethane)^64,65^ and [Cu(bpp)_2_(BF_4_)_2_]_*n*_ (bpp = 1,3-bis(4-pyridyl)propane).^66,67^ From a crystal engineering perspective, if the anionic species are replaced from monovalent (terminal) to divalent pillaring inorganic anions (*e.g.*, SiF_6_^2−^ or NbOF_5_^2−^), 2D CPs can be obtained. For example, bent linkers (*e.g.*, bpe) can afford 2D architectures wherein one direction is spiro-linked, as exemplified by [Zn(SiF_6_)(bpe)_2_]_*n*_^63^ or [Cu(NbOF_5_)(bpe)_2_]_*n*_.^9^ In an effort to pursue this combination of intrinsic porosity and flexibility, we employed a bent bisimidazole linker, 1,3-bis(1*H*-imidazol-1-yl)benzene (bib), to study its potential to form spiro-linked (**SP**) CPs. Specifically, we investigated the effect of metal cations by using CuSiF_6_ and ZnSiF_6_ as reagents. The resulting CPs were found to be 1D structures, where the SiF_6_^2−^ anions occupy pore space as extra-framework counterions. As reported herein, we present an example of the relationship between metal substitution and polymorphism in 1D CPs, and explore how this affects functionality.

## Results and discussion

### Synthesis

Single crystals of **[SP-1-Cu-2MeOH][SIFSIX]-A** and **[SP-1-Zn-2MeOH][SIFSIX]-A** were obtained by layering a methanolic solution of 1,3-bis(1*H*-imidazol-1-yl)benzene (bib, 1) onto aqueous solutions of CuSiF_6_ or ZnSiF_6_, respectively (see SI for details). Whereas the reaction with ZnSiF_6_ afforded a single type of colourless crystals, the reaction with CuSiF_6_ resulted in two types of crystals with light blue and dark blue colours (Fig. S1). Single crystal X-ray diffraction (SCXRD) studies revealed that the two phases obtained in the reaction with CuSiF_6_ were **[SP-1-Cu-2MeOH][SIFSIX]-A** (light blue plates) and **[SP-1-Cu-H_2_O][SIFSIX]-B** (dark blue cubes) (Fig. 1). Crystal packings **A** and **B** differ in the relative arrangement of 1D chains, and are further detailed in the polymorphism section. The two metal variants with **A** packing, **[SP-1-Cu-2MeOH][SIFSIX]-A** and **[SP-1-Zn-2MeOH][SIFSIX]-A**, crystallised in the monoclinic space group *C*2/*c*. Their unit cell parameters are comparable, indicating isostructural CPs, with similar unit cell volumes, namely 2967.00(17) Å^3^ for **[SP-1-Cu-2MeOH][SIFSIX]-A** and 2971.5(4) Å^3^ for **[SP-1-Zn-2MeOH][SIFSIX]-A** (Tables S1 and S2). The asymmetric unit comprises half a metal ion (M = Cu(ii) or Zn(ii)), one coordinated methanol (MeOH) molecule, one bib linker and half a SiF_6_^2−^ (**SIFSIX**) ion. MeOH molecules of solvation are also present in the pores, affording the formula {[M(bib)_2_(MeOH)_2_][SiF_6_]·*x*MeOH}_*n*_ where M = Cu(ii) or Zn(ii). Each bib linker connects two metal centres creating a spiro-loop (**SP**) involving two metals and two linkers (Fig. 1) that in turn generates ribbon-type 1D chains.^40^ The metal ions adopt octahedral coordination geometry with axial sites occupied by MeOH while the nitrogen atoms from bib linkers lie at the equatorial sites. Jahn–Teller distortion^68^ is evident in the case of the Cu(ii) variant (Cu–O distance of 2.405 Å) compared to the Zn(ii) variant (Zn–O distance of 2.176 Å).

**Fig. 1:**
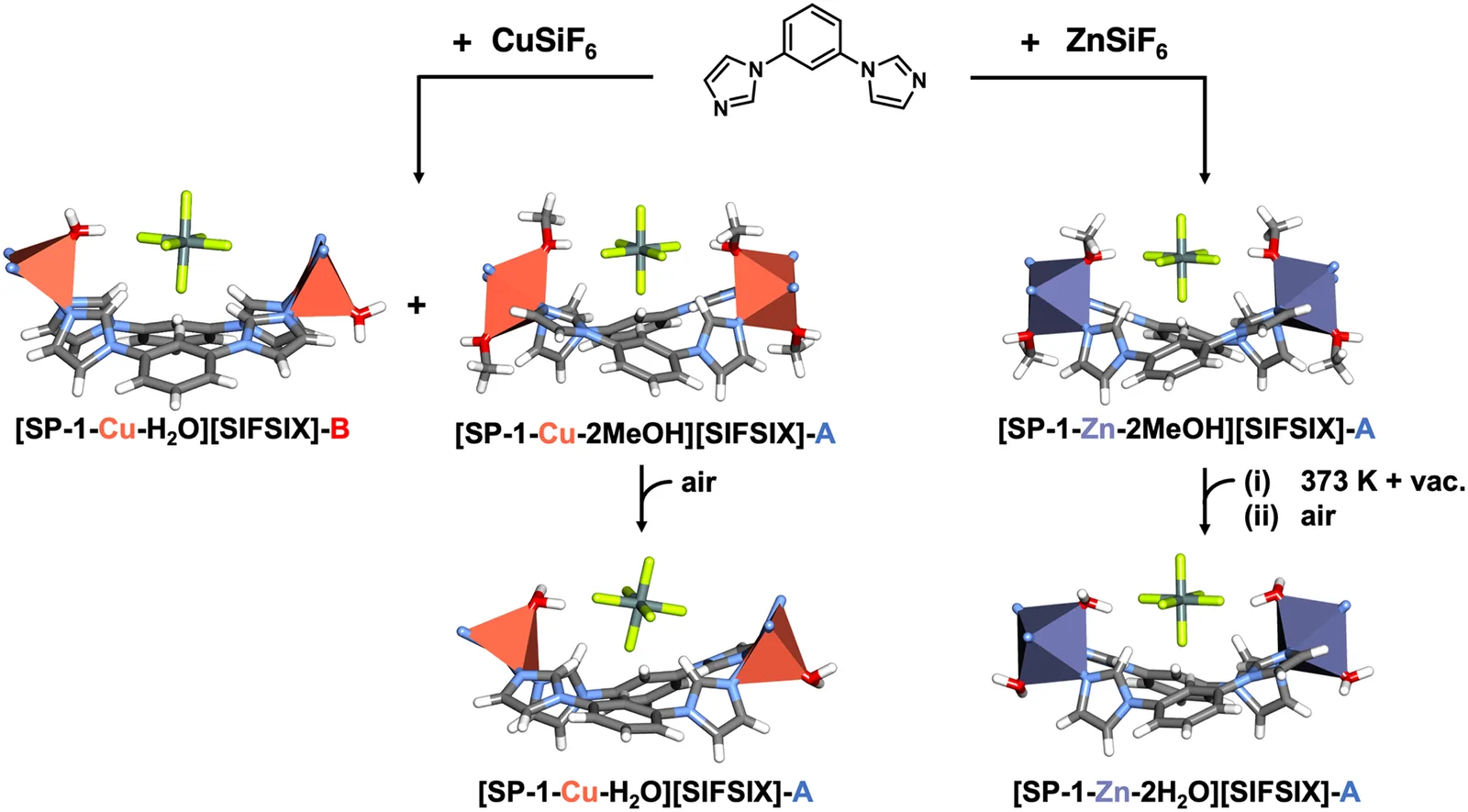
Schematic diagram for the synthesis of CPs based on bib and CuSiF_6_ (left) or ZnSiF_6_ (right). Cu(ii) leads to crystallization of two packing modes, **[SP-1-Cu-H_2_O][SIFSIX]-B** and **[SP-1-Cu-2MeOH][SIFSIX]-A**, whereas Zn(ii) produces a pure phase of **[SP-1-Zn-2MeOH][SIFSIX]-A**. Structural transformations allowed us to isolate **[SP-1-Cu-H_2_O][SIFSIX]-A** from **[SP-1-Cu-2MeOH][SIFSIX]-A**, and [**SP-1-Zn-2H_2_O][SIFSIX]-A** from **[SP-1-Zn-2MeOH][SIFSIX]-A**. Atom colour codes: C, grey; N, blue; O, red; H, white; Si, dark green; F, lime green; Cu, orange; Zn, dark purple. Transition metal atoms are represented by polyhedra.

In both Cu(ii) and Zn(ii) compounds, the SiF_6_^2−^ ions are not coordinated, but rather lie between two MeOH ligands of two different metal sites. They engage in hydrogen bonding with the MeOH ligands, with F⋯O distances of 2.638(3) Å for Cu(ii) and 2.584(4) Å for Zn. In this case, there is no intrinsic porosity because the SiF_6_^2−^ ions occupy the spaces within the spiro loops (Fig. 2a and b), while remaining positioned away from the extrinsic pores. That SiF_6_^2−^ is uncoordinated is perhaps surprising, as related bent linkers afforded pillaring of the anions and higher dimensionality (usually 2D) CPs.^9,44,63^ Nevertheless, a CSD search for SiF_6_^2−^ (version 2025.3.1) in the 1D MOF subset revealed 52 hits, out of which only 13 have the anionic species coordinated (Table S4). Most of the entries are based on Cu(ii), possibly due to the commercial availability of CuSiF_6_. Notably, one of the entries is based on a similar bent ligand, 1,3-di(4*H*-1,2,4-triazol-4-yl)benzene, and Cd(ii).^69^

**Fig. 2:**
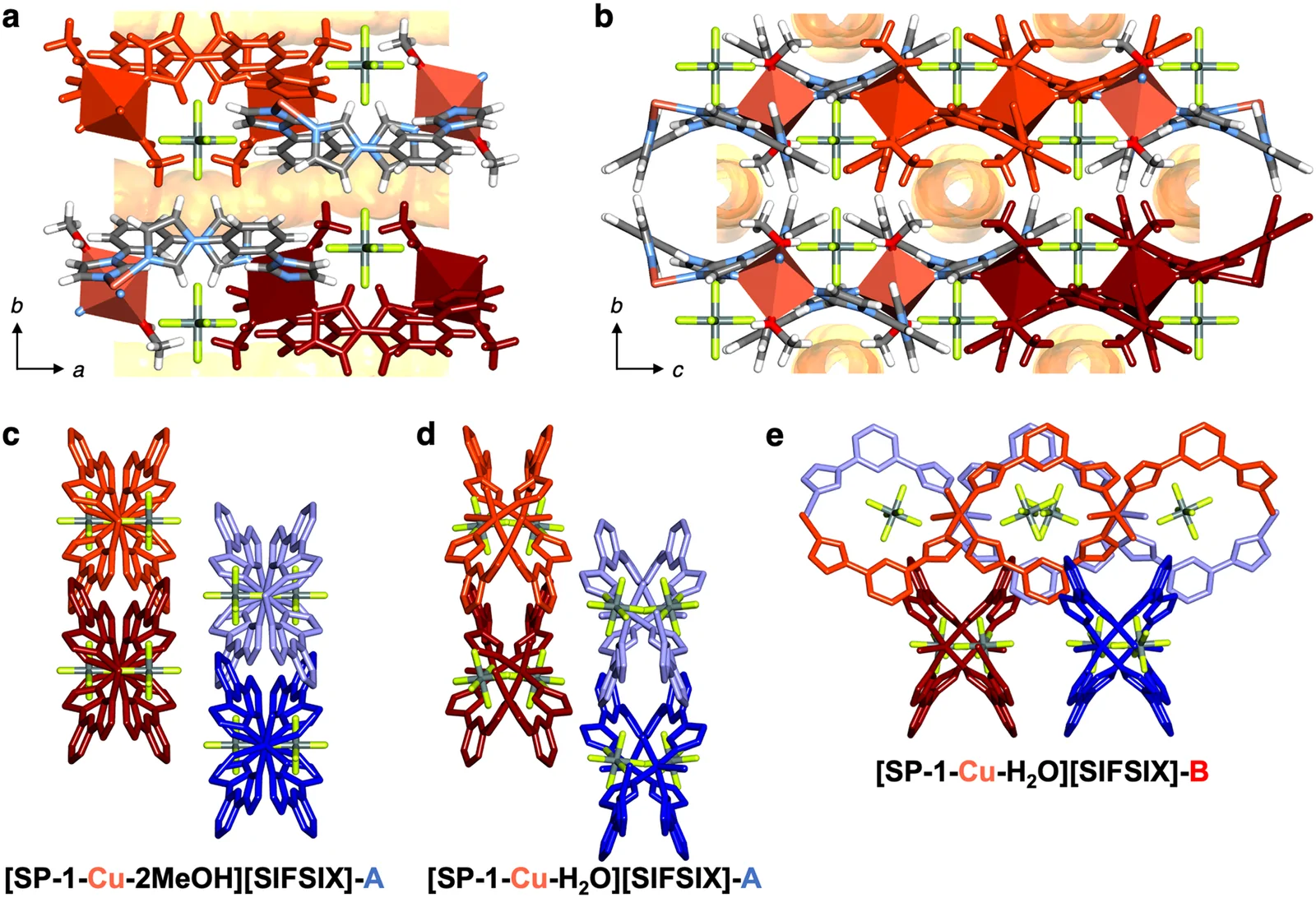
Void space (determined with a probe radius of 1.4 Å) in **[SP-1-Cu-2MeOH][SIFSIX]-A** along the crystallographic (a) *c* axis and (b) *a* axis. Crystal packing in **[SP-1-Cu-2MeOH][SIFSIX]-A** (c), **[SP-1-Cu-H_2_O][SIFSIX]-A** (d) and **[SP-1-Cu-H_2_O][SIFSIX]-B** (e). Atom colour codes: C, grey; N, blue; O, red; H, white; Si, dark green; F, lime green; Cu, orange. Different colours (red, orange, blue, purple) indicate different 1D chains. Hydrogen atoms are omitted for clarity in (c–e). Transition metal atoms are represented by polyhedra in (a–b).

SCXRD revealed that **[SP-1-Cu-H_2_O][SIFSIX]-B** had crystallised in the tetragonal space group *I*4_1_*cd* (Table S3). The asymmetric unit is doubled compared to the **A** packing analogues, with one Cu(ii) ion, one coordinated water molecule, two bib linkers and one SiF_6_^2−^ ion. The asymmetric unit is completed with lattice water molecules, resulting in the composition {[Cu(bib)_2_(H_2_O)][SiF_6_]·*x*H_2_O}_*n*_. The Cu(ii) centre adopts square pyramidal geometry, with a Cu–O distance involving the aqua ligand of 2.185 Å. The aqua ligand is involved in hydrogen bonding with both the water of hydration (O⋯O = 2.76(1) Å) and the SiF_6_^2−^ ions (O⋯F = 2.597(9) Å). The same SiF_6_^2−^ is also involved in additional H-bonds with O⋯F distances of 2.754(9) Å. For single crystals, under the conditions examined, Cu(ii) affords two packing arrangements, whereas only packing **A** was observed for Zn(ii). Additional Zn(ii) polymorphs may, however, exist under conditions not investigated here. For bulk preparation, obtaining pure **[SP-1-Cu-H_2_O][SIFSIX]-A** or **[SP-1-Cu-2MeOH][SIFSIX]-B** was challenging because both crystallised from the same reaction mixture. Since **[SP-1-Cu-H_2_O][SIFSIX]-B** is a hydrate, eliminating water from the reaction afforded pure **[SP-1-Cu-2MeOH][SIFSIX]-A**, as indicated by powder X-ray diffraction (PXRD) (Fig. S2). All attempts to produce **[SP-1-Cu-H_2_O][SIFSIX]-B** in bulk failed, even when water was used as the only solvent medium, forming a powder that was neither of the two polymorphs. Similarly, using a 1 : 1 ratio of the Cu(ii) and Zn(ii) precursors resulted in a product that matched neither the **A** nor the **B** packing (Fig. S3). Nevertheless, calculated PXRD patterns revealed that **[SP-1-Zn-2MeOH][SIFSIX]-A** is isostructural to **[SP-1-Cu-2MeOH][SIFSIX]-A**, while the **B** phase is indeed distinct (Fig. S4). Upon obtaining the **A** packing materials of the Cu(ii) and Zn(ii) analogues in bulk, their stability under acidic and basic conditions was examined. PXRD analysis showed that both materials underwent structural changes within 1 day (Fig. S5 and S6), suggesting their limited stability under these conditions, potentially due to the presence of untethered SiF_6_^2−^ ions.

### Phase transformations

Upon exposure to air, crystals of **[SP-1-Cu-2MeOH][SIFSIX]-A** spontaneously changed colour to a deeper shade of blue within 10 minutes (Fig. S7). Interestingly, this colour was similar to that of **[SP-1-Cu-H_2_O][SIFSIX]-B**, indicating a probable change in the coordination sphere of Cu(ii). Indeed, when a single crystal of **[SP-1-Cu-2MeOH][SIFSIX]-A** was left in the laboratory atmosphere for 20 minutes and analyzed by SCXRD, a new phase, **[SP-1-Cu-H_2_O][SIFSIX]-A**, was identified (Fig. 3). The Cu(ii) centres had adopted square pyramidal coordination geometry with an aqua ligand at the axial position, as seen in **[SP-1-Cu-H_2_O][SIFSIX]-B**. Therefore, two of the coordinated MeOH molecules had been replaced with one aqua ligand. The unit cell volume was reduced by 7.2% (from 2967.00(17) to 2754.1(6) Å^3^). The most notable structural differences were found in the *β* angle (97.3° to 90.9°) and the location of the SiF_6_^2−^ ions. Along the crystallographic *a* axis, the SiF_6_^2−^ moieties rotate by ∼9°, with Si–F⋯O angles between the coordinated oxygen and H-bonded fluorine changing from 121.8° to 130.9° (Fig. 3a). This rotation is also enabled by different N–N–C–C torsion angles in the linker bib, from 13.7(8)° and 54.0(8)° in **[SP-1-Cu-2MeOH][SIFSIX]-A** to 22(3)/23(3)° and 48(3)/52(2)° in **[SP-1-Cu-H_2_O][SIFSIX]-A**.

**Fig. 3:**
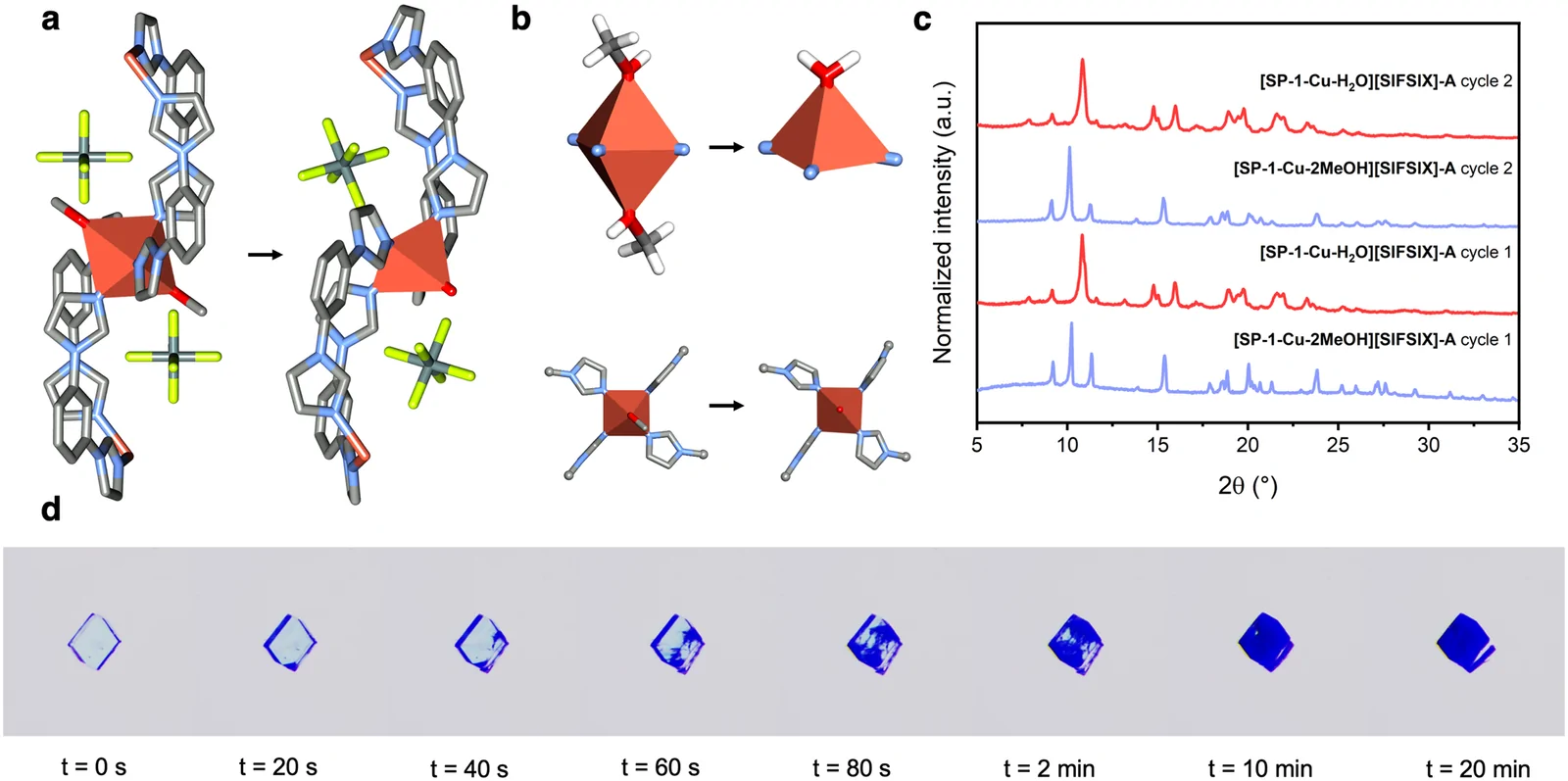
Structural changes upon phase transformation of **[SP-1-Cu-2MeOH][SIFSIX]-A** to **[SP-1-Cu-H_2_O][SIFSIX]-A** in the 1D chain along the crystallographic *a* axis (a) and coordination sphere (b). (c) PXRD patterns for two cycles of transformation between **[SP-1-Cu-2MeOH][SIFSIX]-A** and **[SP-1-Cu-H_2_O][SIFSIX]-A**. (d) Variable-time (*t* = time) optical micrographs of a crystal of **[SP-1-Cu-2MeOH][SIFSIX]-A** exposed to air for 20 minutes. Transition metal atoms are represented by polyhedra in (a–b) and hydrogen atoms are omitted for clarity. Atom colour codes: C, grey; N, blue; O, red; Si, dark green; F, lime green; Cu, orange.

This phase transformation was monitored by PXRD, optical microscopy, and Fourier-transformed infrared spectroscopy (FTIR). First, a PXRD experiment of **[SP-1-Cu-2MeOH][SIFSIX]-A** drying in laboratory atmosphere for 10 minutes revealed transformation to **[SP-1-Cu-H_2_O][SIFSIX]-A** (Fig. 3c). A second cycle, involving addition of one drop of MeOH followed by subsequent drying, showed that the transformation is reversible. Second, optical microscopy of a single crystal of **[SP-1-Cu-2MeOH][SIFSIX]-A** drying in air provided information concerning the process of the phase transformation (Fig. 3d). After 80 s of drying, more than half of the crystal had visibly undergone phase transformation, and, eventually, after 20 minutes of drying, part of the crystal had fractured, indicating that strain associated with this transformation leads to fragmentation into smaller domains. Third, to monitor the MeOH and water content of the two phases, an FTIR experiment was conducted upon drying in air for 10 minutes (Fig. S8). After drying for 5 minutes, peaks corresponding to the O–H stretching of MeOH molecules moved to higher wavenumbers (3370 to 3381 cm^−1^), as would be expected for changes from intramolecular to intermolecular bonding. Further drying for an additional 5 minutes afforded a shift to 3403 cm^−1^. In comparison, **[SP-1-Zn-2MeOH][SIFSIX]-A** showed O–H stretching at a lower wavenumber, 3265 cm^−1^. This is consistent with the stronger (shorter) M–O bonds, which in turn weaken the O–H bonds causing their vibrations to appear at lower wavenumbers.

On the other hand, exposure of **[SP-1-Zn-2MeOH][SIFSIX]-A** to laboratory atmosphere did not afford a similar structural transformation. SCXRD revealed that **[SP-1-Zn-2MeOH][SIFSIX]-A** retained its coordinated MeOH even when exposed to air for 1 day. However, when activated at 373 K under vacuum and subsequent exposure to air for SCXRD analysis, a phase transformation to **[SP-1-Zn-2H_2_O][SIFSIX]-A** was observed (Table S2). The new phase exhibited unit cell parameters consistent with as-synthesised **[SP-1-Zn-2MeOH][SIFSIX]-A**. However, a key difference was found in the coordination sphere of Zn(ii), wherein the two MeOH molecules had been replaced by two aqua ligands, maintaining octahedral geometry. The Zn–O bonds remained at equivalent length (2.169 Å compared to 2.176 Å), displaying similar hydrogen bonding (F⋯O distance of 2.603(4) Å compared to 2.584(4) Å).

### Polymorphism

If we consider the different solvents in **[SP-1-Cu-H_2_O][SIFSIX]-A** and **[SP-1-Cu-H_2_O][SIFSIX]-B**, then the two compounds can be called pseudopolymorphs.^11^ Although the coordination environment is square pyramidal in both, **[SP-1-Cu-H_2_O][SIFSIX]-A** is closer to ideal square pyramidal geometry (Table S5), as **[SP-1-Cu-H_2_O][SIFSIX]-B** deviates from the N–Cu–O angles in the range of 91.8(3)–105.0(3)° instead of 90°. Further, calculation of their densities (Table S6) excluding solvent revealed that **[SP-1-Cu-H_2_O][SIFSIX]-A** (*ρ* = 1.553 g cm^−3^) has a higher density than **[SP-1-Cu-H_2_O][SIFSIX]-B** (*ρ* = 1.499 g cm^−3^). **[SP-1-Cu-H_2_O][SIFSIX]-A** is the denser phase and would be expected to be the most stable pseudopolymorph. This is consistent with our ability to reliably synthesise **[SP-1-Cu-2MeOH][SIFSIX]-A** (*ρ* = 1.545 g cm^−3^). The different 1D chains are connected by weak intermolecular forces in both pseudopolymorphs, with SiF_6_^2−^ ions playing a crucial role. Specifically, the SiF_6_^2−^ anion species lie between chains, engaging in weak C–H⋯F interactions (Fig. 2).^70^ In **[SP-1-Cu-H_2_O][SIFSIX]-A**, the same anion interacts with four adjacent chains, with C–H⋯F contacts of 3.00(1), 3.05(1), 3.08(1) and 3.10(1) Å. Conversely, in **[SP-1-Cu-H_2_O][SIFSIX]-B**, four C–H⋯F contacts interact with perpendicular chains (3.03(1), 3.19(1), 3.21(1) and 3.50(1) Å), while two C–H⋯F interactions connect parallel chains (3.01(1) and 3.06(1) Å). Nevertheless, these interactions are relatively weak and the SiF_6_^2−^ species are mobile in the absence of coordination, raising questions about the activation and stability of these materials.

### Activation and properties

The different behaviour of **[SP-1-Cu-2MeOH][SIFSIX]-A** and **[SP-1-Zn-2MeOH][SIFSIX]-A** upon exposure to laboratory atmosphere is noteworthy. Although both materials adopt the same packing polymorph **A**, their difference in metal affords distinct properties. Whereas Cu(ii) underwent transformation from octahedral to square pyramidal geometry, Zn(ii) remained octahedral. In essence, this results in the final phases having one (for Cu(ii)) or two (for Zn(ii)) aqua ligands. The driving force behind the facile transformation of **[SP-1-Cu-2MeOH][SIFSIX]-A** to **[SP-1-Cu-H_2_O][SIFSIX]-A** might be explained by examination of hydrogen bond donor/acceptor maps (Fig. 4a). As seen on the map of the Cu(ii) variant, the aqua ligands provide more hydrogen-bonding interactions than MeOH. This is expected since the aqua ligands have two hydrogen atoms and are therefore involved in one additional hydrogen bond with a nearby MeOH molecule (O⋯O 2.80(1) Å) in **[SP-1-Cu-H_2_O][SIFSIX]-A**. Similarly, a higher number of hydrogen bonds are observed in **[SP-1-Zn-2H_2_O][SIFSIX]-A** compared to **[SP-1-Zn-2MeOH][SIFSIX]-A** (Fig. 4a). However, the absence of a second aqua ligand in **[SP-1-Cu-H_2_O][SIFSIX]-A** required further analysis. To investigate this, a CSD survey of the M–O bond length in coordination compounds containing M = Cu(ii) or Zn(ii) was conducted (Fig. 4b). The coordination number was specified to 5 and 6 for each metal, to investigate the effect of the coordination sphere on the M–O bonds. The results revealed that, while Zn–O bond lengths are mostly independent of coordination number, Cu–O bonds are noticeably shorter in 5-coordinate complexes than 6-coordinate examples. The facile conversion of octahedral to square pyramidal geometries for Cu^2+^ arises from Jahn–Teller distortion of the d^9^ configuration, which significantly weakens the axial bonds, presumably making ligand dissociation energetically accessible. In contrast, Zn(ii) does not exhibit the same Jahn–Teller-driven preference for reduced coordination, and the octahedral environment is therefore retained. Consequently, Zn(ii) affords only the diaqua environment, whereas the square-pyramidal **B** packing is not observed.

**Fig. 4:**
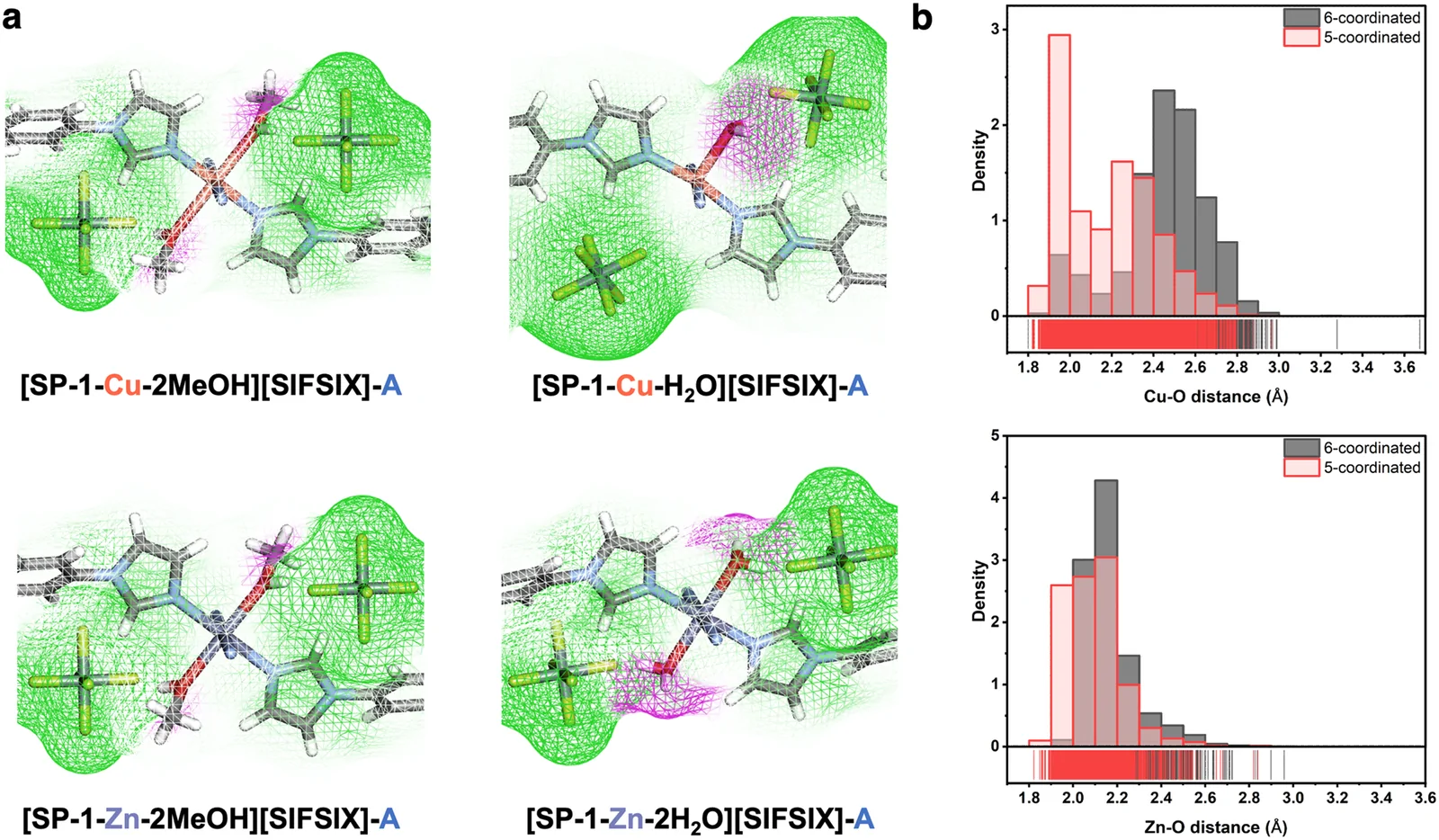
(a) Hydrogen bond maps for **[SP-1-Cu-2MeOH][SIFSIX]-A**, **[SP-1-Cu-H_2_O][SIFSIX]-A**, **[SP-1-Zn-2MeOH][SIFSIX]-A** and **[SP-1-Zn-2H_2_O][SIFSIX]-A** (pink = H-bond donor, green = H-bond acceptor). (b) Outcome of a CSD survey on the Cu–O (top) or Zn–O (bottom) distances in 5-coordinated (red) and 6-coordinated (grey) coordination environments. Atom colour codes: C, grey; N, blue; O, red; H, white; Si, dark green; F, lime green; Cu, orange; Zn, dark purple.

After gaining some understanding concerning the driving force behind the flexibility of **[SP-1-Cu-2MeOH][SIFSIX]-A** compared to the relative rigidity of **[SP-1-Zn-2MeOH][SIFSIX]-A**, we attempted to activate these materials for gas sorption studies. To this end, variable-temperature PXRD (VT-PXRD) experiments were first conducted under vacuum to assess stability to heat and vacuum. **[SP-1-Zn-2H_2_O][SIFSIX]-A** remained stable under vacuum and heating up to 423 K, as indicated by sharp PXRD peaks matching the initial phase (Fig. 5a). On the other hand, neither **[SP-1-Cu-H_2_O][SIFSIX]-A** nor **[SP-1-Cu-2MeOH][SIFSIX]-A** was found to be stable to vacuum at room temperature, with loss of crystallinity after 15 minutes of exposure to vacuum (Fig. 5b). This instability arising from a simple metal substitution in otherwise isostructural compounds is unusual, as CPs are often studied as families in which properties are fine-tuned by crystal engineering strategies, *e.g.* metal substitution. However, in retrospect, the greater stability of **[SP-1-Zn-2H_2_O][SIFSIX]-A** may arise from the additional aqua ligand, offering a higher number of interactions with the SiF_6_^2−^ species, effectively anchoring them in place.

**Fig. 5:**
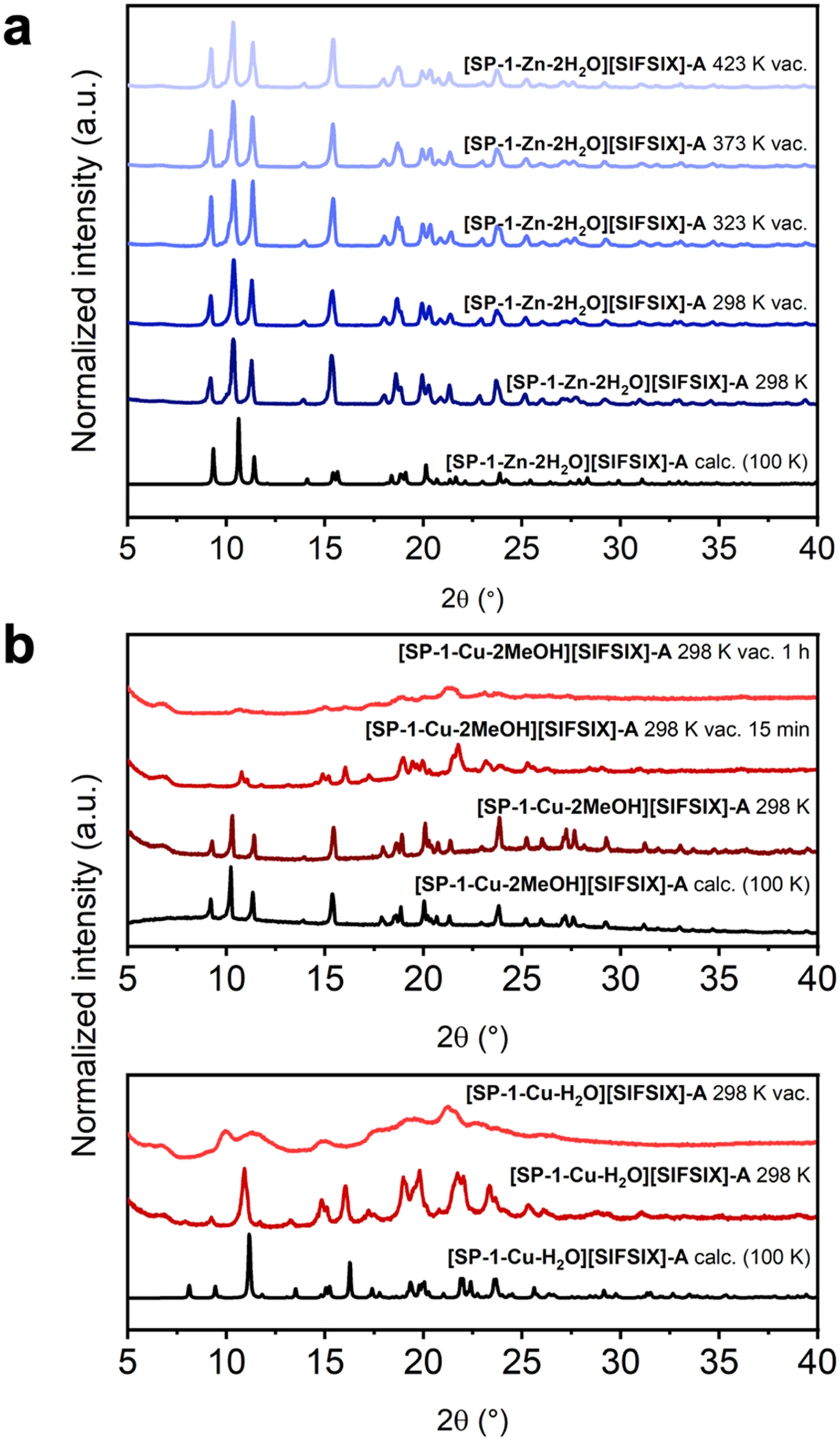
(a) VT-PXRD experiment under vacuum from 298 to 423 K for **[SP-1-Zn-2H_2_O][SIFSIX]-A**. (b) PXRD experiments under vacuum at 298 K for **[SP-1-Cu-2MeOH][SIFSIX]-A** (top) and **[SP-1-Cu-H_2_O][SIFSIX]-A** (bottom).

To further investigate the relatively stable phase, **[SP-1-Zn-2H_2_O][SIFSIX]-A**, we explored its potential to adsorb gases or water vapour. Thermogravimetric analysis (TGA) showed that the loss of solvated MeOH occurred by 333 K, while the aqua ligands remained up to 423 K (Fig. S9). Further, differential scanning calorimetry (DSC) revealed no phase transformations up to this temperature, suggesting that no structural transformation had occurred during removal of guest molecules (Fig. S10). The second DSC cycle revealed two endothermic peaks above 450 K, indicating more facile decomposition upon guest removal. CO_2_ gas sorption isotherms were then collected at 195 and 298 K, showing weak type I isotherms (Fig. 6 and S11). The uptake at 298 K and 1 bar was 14.7 cm^3^ g^−1^, which corresponds to only *ca.* 0.5 molecule of CO_2_ per Zn (Table S7). We also investigated the water vapour sorption properties of **[SP-1-Zn-2H_2_O][SIFSIX]-A**, which revealed weakly hydrophilic adsorption isotherms (Fig. 6 and S12). The uptake at 90% relative humidity (RH) was 6.0 wt%, which corresponds to the adsorption of two water molecules per Zn (Table S8). This approximately fourfold increase in uptake compared to CO_2_ (*V*_g_ = 34 Å^3^) could possibly be ascribed not only to the smaller size of H_2_O (*V*_g_ = 19 Å^3^) but also to hydrogen bonding with H_2_O promoted by the coordinated aqua ligands. Overall, these adsorption properties support the relatively higher structural robustness of the Zn(ii) analogue compared to the Cu(ii) materials.

**Fig. 6:**
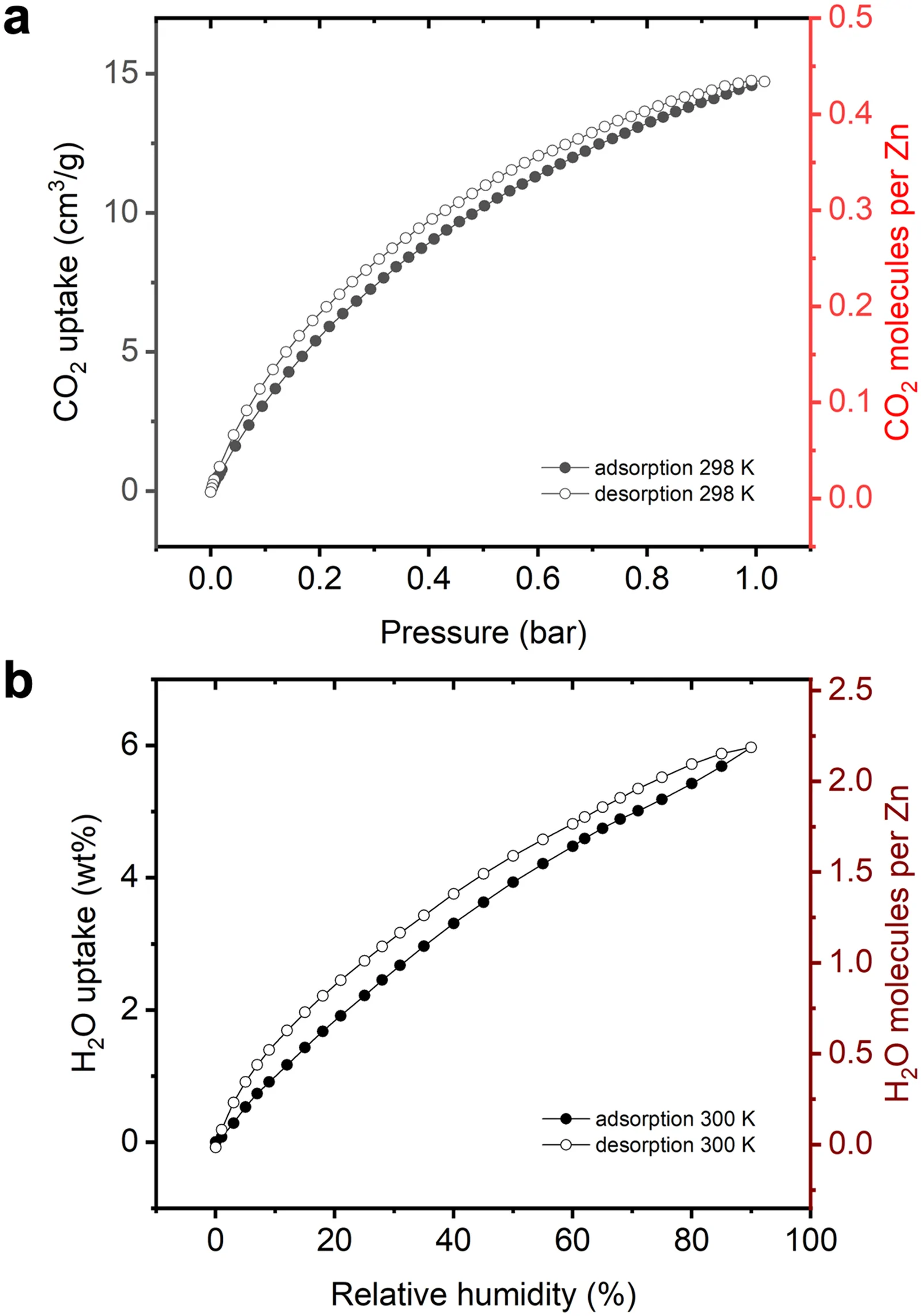
Sorption isotherms for **[SP-1-Zn-2H_2_O][SIFSIX]-A** for (a) CO_2_ at 298 K and (b) H_2_O at 300 K. Full circles = adsorption; open circles = desorption.

We next investigated the possibility of removing the aqua ligands in **[SP-1-Zn-2H_2_O][SIFSIX]-A**, since Zn(ii) may adopt tetrahedral coordination geometry. We hypothesised that coordination of the (SiF_6_^2−^) anions upon aqua ligand removal might occur, as demonstrated for the elastic layered material (ELM) family.^71^ For example, a similar transformation occurs from [Cu(bpy)(BF_4_)_2_(H_2_O)_2_·(bpy)]_*n*_ (**pre-ELM-11**; bpy = 4,4′-bipyridine) to [Cu(bpy)_2_(BF_4_)_2_]_*n*_ (**ELM-11**) upon exposure to heat and vacuum. In the case of **[SP-1-Zn-2H_2_O][SIFSIX]-A**, the SiF_6_^2−^ anions are 4.06 Å away from the Zn(ii) centre, which is similar to the distance (4.07 Å) between Cu(ii) and the nitrogen atom of bpy in the aqua precursor **pre-ELM-11**.^72^ Since SiF_6_^2−^ is potentially ditopic, a second coordination bond would need to be created with an adjacent metal center for a higher dimensionality solid. The nearest Zn(ii) centre of a different chain in **[SP-1-Zn-2H_2_O][SIFSIX]-A** is 6.89 Å, which is generally too far for a solid-state transformation. TGA indicated that release of the two aqua ligands started at 423 K, with a weight loss of 9.5 wt% matching the expected mass of two water molecules (Fig. S9). However, upon heating at 448 K under vacuum for one hour, loss of crystallinity was observed by PXRD (Fig. S13), indicating that the material is physically unstable when the two aqua ligands are removed. Nevertheless, the material retained some crystallinity after being stored in air for 1 year, as indicated by PXRD (Fig. S14). Overall, although the gas and vapour sorption properties of **[SP-1-Zn-2H_2_O][SIFSIX]-A** are ordinary, the ability to fine-tune flexibility and packing polymorphism in this family is relatively uncommon, especially in 1D CPs.^40^

## Conclusions

In conclusion, we report a family of 1D CPs that exhibit packing polymorphism and phase transformations upon exposure to air, heat and vacuum. Whereas **[SP-1-Cu-2MeOH][SIFSIX]-A** is flexible and undergoes a structural transformation to **[SP-1-Cu-H_2_O][SIFSIX]-A**, the Zn(ii) variant **[SP-1-Zn-2MeOH][SIFSIX]-A** is rigid and activates to **[SP-1-Zn-2H_2_O][SIFSIX]-A**. As a result of this flexibility, **[SP-1-Cu-2MeOH][SIFSIX]-A** was found to be unstable to vacuum and heat, while **[SP-1-Zn-2MeOH][SIFSIX]-A** was successfully activated using heat, vacuum, or nitrogen flow. The activated phase of **[SP-1-Zn-2H_2_O][SIFSIX]-A** exhibited type I isotherms for CO_2_ (195 and 298 K) and water vapour (300 K). Further, **[SP-1-Cu-H_2_O][SIFSIX]-A** is a packing polymorph of **[SP-1-Cu-H_2_O][SIFSIX]-B**, which had crystallised concomitantly from the same reaction. Not only could **[SP-1-Cu-H_2_O][SIFSIX]-A** be synthesized by tuning the reaction conditions, but changing the metal to Zn(ii) afforded only the **A** packing polymorph, **[SP-1-Zn-2MeOH][SIFSIX]-A**. These results suggest that metal substitution may influence polymorph formation in 1D CPs through differences in coordination environment, and that it can impact the relative stability of the obtained CPs. We find that this behaviour arises from the coordination geometry and the suitability of the metal to adopt the desired structural arrangement.

## Author contributions

Kyriaki Koupepidou: conceptualization; investigation; formal analysis; visualization; writing – original draft; writing – review & editing. Asif Raza, Tao He: resources; writing – review & editing. Alan C. Eaby: investigation; writing – review & editing. Soumya Mukherjee: conceptualization; funding acquisition; writing – review & editing. Michael J. Zaworotko: conceptualization; supervision; funding acquisition; writing – review & editing.

## Conflicts of interest

There are no conflicts to declare.

## Supplementary Material

CE-OLF-D6CE00723F-s001

## Data Availability

The data supporting this article are included as part of the supplementary information (SI). Supplementary information: methods, supplementary figures and tables. See DOI: https://doi.org/10.1039/d6ce00723f. CCDC 2557190–2557194 contain the supplementary crystallographic data for this paper.^73*a*–*e*^
