# Supplementary material for: Effect of metal substitution on polymorphism and flexibility of 1D coordination polymers
Source: CrystEngComm. 2026 Nov 5. Online ahead of print. doi: 10.1039/d6ce00723f (PMC13735481; doi:10.1039/d6ce00723f)
Supplement: Supplementary file 1 [file CE-OLF-D6CE00723F-s001.pdf]

Supplementary Information (SI) for CrystEngComm.
This journal is © The Royal Society of Chemistry

Supplementary Information

Effect of metal substitution on polymorphism and flexibility of 1D coordination polymers

Kyriaki Koupepidou,[†‡] Asif Raza,[†] Tao He,[†] Alan C. Eaby,[†] Soumya Mukherjee[†] and Michael J. Zaworotko[†]**

[†] Department of Chemical Sciences, Bernal Institute, University of Limerick, Limerick V94T9PX, Republic of Ireland

[‡] Institute for Integrated Cell-Material Sciences, Kyoto University Institute for Advanced Study, Kyoto University, Yoshida Ushinomiya-cho, Sakyo-ku, Kyoto, 606-8501 Japan

S1. Materials and methods

S1.1 Materials and synthesis

The linker 1,3-bis(imidazol-1-yl)benzene (bib) was synthesised with modified reported procedures.[1] The salts $CuSiF_6 \cdot 4H_2O$ and $ZnSiF_6 \cdot 6H_2O$ were synthesised according to reported procedures.[2, 3] Other reagents and solvents were commercially available and were used without further purification.

Synthesis of **[SP-1-Cu-2MeOH][SIFSIX]-A** ($\{[Cu(bib)_2(MeOH)_2][SiF_6] \cdot x MeOH\}_n$). Single crystals were obtained by layering a solution of bib (11 mg, 0.05 mmol) in 2 mL of methanol on top of a solution of $CuSiF_6 \cdot 4H_2O$ (14 mg, 0.05 mmol) in 2 mL of water in a test tube. The layers were left to diffuse for 5 days at room temperature, after which light blue plate crystals were formed, along with **[SP-1-Cu-H$_2$O][SIFSIX]-B** (see below). Bulk synthesis was made by slurry method in MeOH in 5-fold scale, after stirring at room temperature for 5 hours.

Synthesis of **[SP-1-Cu-H$_2$O][SIFSIX]-B** ($\{[Cu(bib)_2(H_2O)][SiF_6] \cdot x H_2O\}_n$). Single crystals of **[SP-1-Cu-H$_2$O][SIFSIX]-B** crystallised as dark blue cubic crystals, in the same test tube as **[SP-1-Cu-2MeOH][SIFSIX]-A**. Bulk synthesis was not possible as slurry in H_2O or H_2O/MeOH produced a different compound.

Synthesis of **[SP-1-Cu-H$_2$O][SIFSIX]-A** ($\{[Cu(bib)_2(H_2O)][SiF_6] \cdot x MeOH\}_n$). Single crystals or the bulk phase of **[SP-1-Cu-2MeOH][SIFSIX]-A** were left to dry in air for 20 minutes to obtain **[SP-1-Cu-H$_2$O][SIFSIX]-A**.

Synthesis of **[SP-1-Zn-2MeOH][SIFSIX]-A** ($\{[Zn(bib)_2(MeOH)_2][SiF_6] \cdot x MeOH\}_n$). Single crystals and bulk of **[SP-1-Zn-2MeOH][SIFSIX]-A** were obtained in the same way as described for **[SP-1-Cu-2MeOH][SIFSIX]-A**, by using $ZnSiF_6 \cdot 6H_2O$ (0.05 mmol) instead of $CuSiF_6 \cdot 4H_2O$.

Synthesis of **[SP-1-Zn-2H$_2$O][SIFSIX]-A** ($\{[Zn(bib)_2(H_2O)_2][SiF_6] \cdot x MeOH\}_n$). Single crystals or the bulk phase of **[SP-1-Zn-2MeOH][SIFSIX]-A** heated under vacuum for 30 minutes at 373 K and then exposed to air to obtain **[SP-1-Zn-2H$_2$O][SIFSIX]-A**.

S1.2 CSD database search

The ConQuest function in the Cambridge Structural Database (CSD)[4] was implemented in order to examine the 1D MOF subset.

S1.3 Single-crystal X-ray Diffraction Measurements

Suitable single crystals of **[SP-1-Cu-2MeOH][SIFSIX]-A**, **[SP-1-Cu-H_2O][SIFSIX]-B**, **[SP-1-Cu-H_2O][SIFSIX]-A**, **[SP-1-Zn-2MeOH][SIFSIX]-A** and **[SP-1-Zn-2H_2O][SIFSIX]-A** were chosen for single-crystal X-ray diffraction measurements. Diffraction data were collected at 150 K on a Bruker D8 Quest diffractometer equipped with a MoKα microfocus source (λ = 0.7107 Å) and a Photon 100 detector. In all cases, data was indexed, integrated and scaled in APEX4. Absorption correction was performed by multi-scan method using in SADABS. Space group determination was performed simultaneously with structure solution using SHELXT intrinsic phasing methods and the solution was refined on F2 using SHELXL non-linear least squares implemented in Olex2 v1.2.10. Anisotropic thermal parameters were applied to all non-hydrogen atoms. All the hydrogen atoms were generated geometrically. X-ray experimental data and refinement parameters are given in Tables S1–S3. The final crystal structures have been deposited in the Cambridge Crystallographic Data Centre (CCDC 2557190-2557194). Maps shown in Figure 4 of the main manuscript were constructed using the hydrogen bond donor/acceptor surface function in BIOVIA Discovery Studio.[5]

S1.4 Powder X-ray Diffraction Measurements

Powder X-ray diffraction data was collected on crushed microcrystalline samples. Diffractograms were recorded using a PANalytical Empyrean™ diffractometer equipped with a PIXcel3D detector, operating in scanning line detector mode with an active length of 4 utilizing 255 channels, in the Continuous Scanning mode with the goniometer in the theta-theta orientation. The diffractometer is fitted with an Empyrean Cu LFF (long fine-focus) HR (9430 033 7310x) tube operated at 40 kV and 40 mA, and CuKα radiation (λα = 1.540598 Å) was used for diffraction experiments. Incident beam optics included the Fixed Divergences slit with anti-scatter slit PreFIX module, with a 1/8° divergence slit and a 1/4° anti-scatter slit, as well as a 10 mm fixed incident beam mask and a Soller slit (0.04 rad). Divergent beam optics included a P7.5 anti-scatter slit, a

Soller slit (0.04 rad), and a Ni-β filter. The data was collected from 4°-40° (2θ) with a step-size of 0.016413° and a varied scan time of 30-200 seconds per step.

S1.5 Variable-Temperature Powder X-ray Diffraction Measurements

Diffractograms at different temperatures were recorded using a PANalytical X'Pert Pro-MPD diffractometer equipped with a PIXcel3D detector, operating in scanning line detector mode with an active length of 4 utilizing 255 channels. Anton Paar TTK 450 stage coupled with the Anton Paar TCU 110 Temperature Control Unit were used to record the variable-temperature diffractograms. The diffractometer was outfitted with an Empyrean Cu LFF (long fine-focus) HR (9430 033 7300x) tube operated at 40 kV and 40 mA and CuKα radiation (λ = 1.54056 Å). Continuous scanning mode with the goniometer in the theta-theta orientation was used to collect the data. Incident beam optics included a 1/4° divergence slit and a Soller slit (0.04 rad). Divergent beam optics included a P7.5 anti-scatter slit, a Soller slit (0.04 rad) and a Ni-β filter. In a typical experiment, *ca.* 20 mg of sample was crushed to microcrystalline powder and was loaded on a zero background sample holder made for the Anton Paar TTK 450 chamber. Each sample was heated up to the noted temperature (no more than 200 ˚C) under N_2 atmosphere and then cooled back to room temperature. The data was collected from 3° to 35° (2θ) with a step-size of 0.016413° and a scan time of 200 seconds per step.

S1.6 Thermogravimetric Analysis (TGA) and Differential Scanning Calorimetry (DSC)

Thermogravimetric analysis (TGA) was performed using a TA Instruments Q50 system. Samples were loaded into aluminium sample pans and heated at 10 K/min from room temperature to 823 K under N_2 flow.

Differential scanning calorimetry (DSC) analysis was performed on a Q2000 TA Instruments system. Samples were loaded into aluminium sample pans with a pinhole on the lids. The experiments were performed at heating rate of 5 °C/min from room temperature up to 473 K under N_2 atmosphere.

S1.7 Fourier Transform Infrared (FTIR) Spectroscopy

Spectra were obtained by using a Perkin Elmer Spectrum 100 FTIR Spectrometer with ATR and Spotlight 200 FTIR microscope attachment. A small amount of powder microcrystalline sample

was placed onto the ATR plate and the spectra were collected in the range of 4000-650 cm^{-1} with resolution of 0.5 cm^{-1}.

S1.8 Gas Sorption Measurements

For gas sorption experiments, high-purity gases were used as received from BOC Gases Ireland: CO_2 (99.995%). A Micromeritics 3Flex surface area and pore size analyser was used. The temperature at 195 K was maintained using a 4 L Dewar filled with a dry ice-acetone mixture. Bath temperature at 298 K was maintained using a 4 L Dewar filled with water. Before each gas sorption experiment, the freshly prepared sample of **[SP-1-Zn-2MeOH][SIFSIX]-A** was placed in a quartz tube and activated under high vacuum at 373 K on a Micromeritics SmartVacPrep for 12 hrs, to generate the activated phase.

S2. Tables and figures

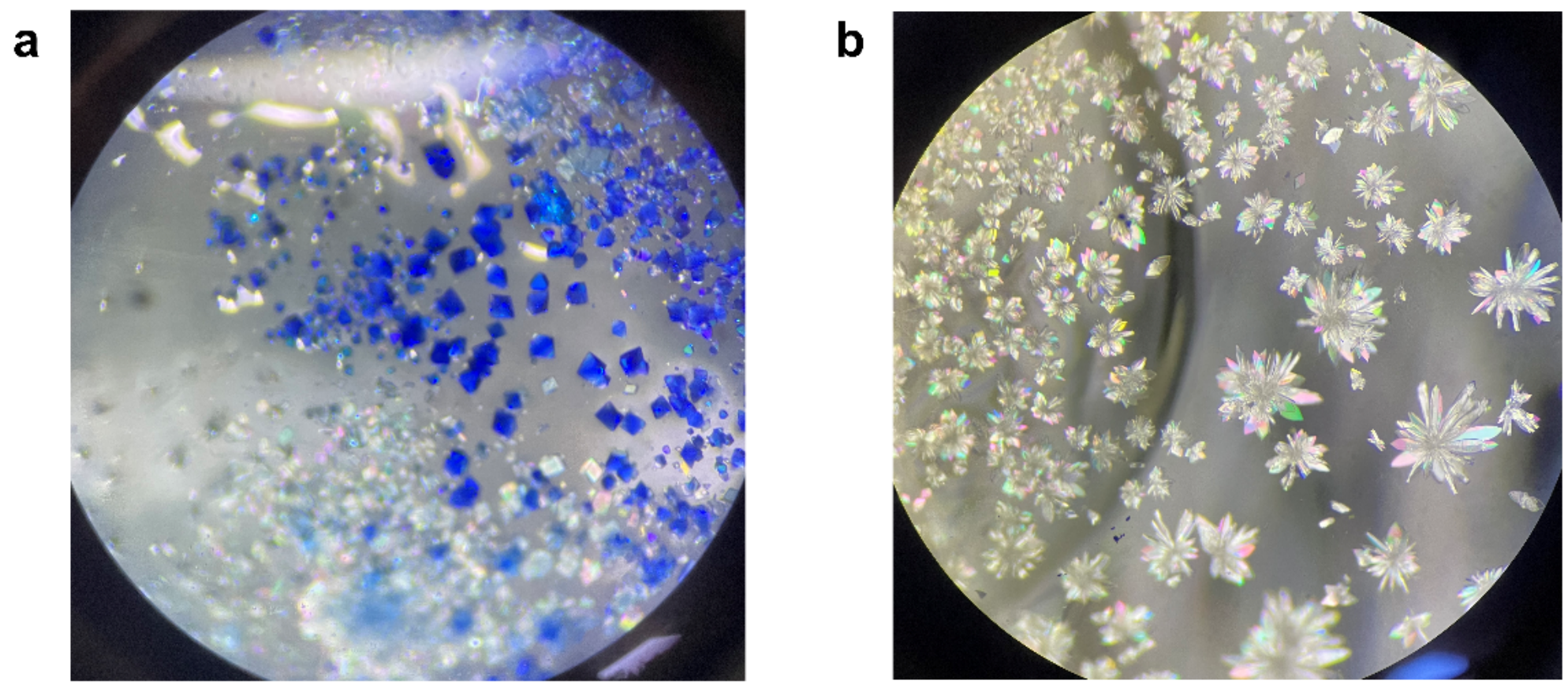

Figure S1. Optical micrographs of crystallization outcomes using bib and (a) $CuSiF_6$ and (b) $ZnSiF_6$ at room temperature.

Table S1. Crystallographic and refinement parameters for **[SP-1-Cu-2MeOH][SIFSIX]-A** and **[SP-1-Cu-H_2O][SIFSIX]-A**.

Compound	[SP-1-Cu-2MeOH][SIFSIX]-A	[SP-1-Cu-H_2O][SIFSIX]-A
Formula	$[C_{13}H_{14}Cu_{0.5}N_4O][Si_{0.5}F_3]\cdot x(CH_3O)$	$[C_{24}H_{21}CuN_8O][SiF_6]\cdot x(CH_4O)$
Formula weight	348.74	675.16
Temperature (K)	150	150
Crystal system	Monoclinic	Monoclinic
Space group	*C2/c*	$P2_1/n$
a (Å)	11.3975(4)	11.5962(15)
b (Å)	16.8813(5)	15.865(2)
c (Å)	15.5483(5)	14.972(2)
α (°)	90	90
β (°)	97.3480(10)	90.882(3)
γ (°)	90	90
V ($Å^3$)	2967.00(17)	2754.1(6)
Z	8	4
D_c ($g\cdot cm^{-3}$)	1.561	1.628
μ (mm^{-1})	0.856	0.918
R_{int}	0.0478	0.2688
GOF	1.048	1.029
R_1 [I > 2σ (I)]	0.0390	0.0930
WR_2 [all data]	0.1051	0.2273
Diff peak / hole (e $Å^{-3}$)	0.909 / -1.501	0.896 / -1.647
No. CCDC	2557190	2557191

Table S2. Crystallographic and refinement parameters for **[SP-1-Zn-2MeOH][SIFSIX]-A** and **[SP-1-Zn-2H$_2$O][SIFSIX]-A**.

Compound	[SP-1-Zn-2MeOH][SIFSIX]-A	[SP-1-Zn-2H$_2$O][SIFSIX]-A
Formula	$[C_{13}H_{14}Zn_{0.5}N_4O][Si_{0.5}F_3]\cdot x(CH_3O)$	$[C_{12}H_{12}Zn_{0.5}N_4O][Si_{0.5}F_3]\cdot x(CH_4O)$
Formula weight	338.93	331.99
Temperature (K)	150	150
Crystal system	Monoclinic	Monoclinic
Space group	*C*2/*c*	*C*2/*c*
a (Å)	11.5686(10)	11.5670(11)
b (Å)	16.6334(13)	16.7434(16)
c (Å)	15.5428(13)	15.6234(14)
α (°)	90	90
β (°)	96.509(2)	96.619(4)
γ (°)	90	90
V (Å^3)	2971.5(4)	3005.6(5)
Z	8	8
D_c (g·cm^{-3})	1.515	1.467
μ (mm^{-1})	0.943	0.930
R_{int}	0.0650	0.0584
GOF	1.048	1.095
R_1 [I > 2σ (I)]	0.0460	0.0529
WR_2 [all data]	0.1488	0.1743
Diff peak / hole (e Å^{-3})	0.554 / -0.646	0.820 / -0.803
No. CCDC	2557192	2557193

Table S3. Crystallographic and refinement parameters for **[SP-1-Cu-H_2O][SIFSIX]-B**.

**Compound**	**[SP-1-Cu-H_2O][SIFSIX]-B**
Formula	$[C_{24}H_{22}CuN_8O][SiF_6]\cdot x(H_2O)$
Formula weight	662.14
Temperature (K)	150
Crystal system	Tetragonal
Space group	$I\,4_1cd$
a (Å)	18.8932(4)
b (Å)	18.8932(4)
c (Å)	31.9832(12)
α (°)	90
β (°)	90
γ (°)	90
V ($Å^3$)	11416.5(6)
Z	16
D_c ($g\cdot cm^{-3}$)	1.541
μ (mm^{-1})	2.185
R_{int}	0.0660
GOF	1.282
R_1 [$I > 2\sigma(I)$]	0.0751
WR_2 [all data]	0.2173
Diff peak / hole (e $Å^{-3}$)	1.332 / -0.557
No. CCDC	2557194

Table S4. CSD (version 2025.3.1) survey of 1D coordination polymers containing SiF_6^{2-} anions. Shaded entries correspond to coordinated SiF_6^{2-} anions. The entry with the refcode XIGLIK is shown in red, and is based on a similar ligand to the one studied herein.

No.	CSD Refcode	Metal	Organic ligand	Reference
**1**	ADAWOS	Ag^{2+}	4,4'-bipyridine	10.1107/S1600536806018150
**2**	ADEFEW	Cu^{2+}	1,4-*m*-bis[bis(1-pyrazolyl)methyl]benzene	10.1002/ejic.201200118
**3**	AKIYOK	Cd^{2+}	*N*-pyridin-3-yl-2-[4-(pyridin-3-ylcarbamoylmethyl)phenyl]acetamide	10.1021/acs.cgd.6b00381
**4**	AKIYUQ		*N*-pyridin-3-yl-2-[4-(pyridin-3-ylcarbamoylmethyl)phenyl]acetamide	10.1021/acs.cgd.6b00381
**5**	AVUZAS	Cu^{2+}	3,3'-azobis(pyridine	10.1016/j.molstruc.2004.01.015
**6**	BAWKOB	Cu^{2+}	2,2'-bipyrimidine	10.1021/ic202740b
**7**	BOHFAF	Co^{2+}	*N*-vinylimidazole	10.1021/ic00140a004
**8**	BUPGEZ	Zn^{2+}	4,4'-(pentane-1,5-diyldisulfanediyl)dipyridine	10.1039/b917864c
**9**	CEDFAV	Cu^{2+}	2-allylamino-1,3-thiazol-4(5*H*)-one	10.1080/00958972.2017.1286012
**10**	COXBUP	Cu^{2+}	*N*,*N'*-(propane-1,3-diyl)bis(1-(1*H*-imidazol-4-yl)methanimine)	10.1039/C9DT04368C
**11**	DAYQED	Cu^{2+}	3-(prop-2-en-1-ylamino)propanenitrile	10.17344/acsi.2016.3116
**12**	DAZHAQ	Zn^{2+}	pyrazine	10.1002/chem.201605507
**13-14**	DEPCOR/ DEPCUX	Cu^{2+}	*N*,*N*'-bis(3-pyridyl)urea	10.1039/b606150h
**15**	EBAVIO	Cu^{2+}	1-allylbenzotriazole	10.1016/j.ica.2011.08.008
**16**	EFUBEO	Cu^{2+}	1,4-di-(1,2,4-triazole-4-yl) benzene	10.1080/15533174.2013.799493

Table S4 continued.

**17**	FEFVIW	Cu^{2+}	pyrazine	10.1002/ejic.200400438
**18-19**	FIZCIE/ FIZCOK	Cu^{2+}	1-(nicotinamido)-2,3-dihydro-1*H*-inden-2-yl nicotinate	10.1021/acsomega.3c05659
**20**	HAGRUF	Cu^{2+}	4-amino-4*H*-1,2,4-triazole	10.3390/ijms141223597
**21-23**	HIPDAM/ HIPDEQ/ HIPDIU	Co^{2+}/ Cu^{2+}/ Zn^{2+}	pyrimidine	10.1021/cg070462p
**24**	HULTAL	Cd^{2+}	3,6-bis (1*H*-imidazol-1-yl)pyridazine	CSD communication
**25**	KAJGIN	Ag^{2+}	*N*,*N*''-ethylene-1,2-diylbis(*N*'-pyridin-3-ylurea)	10.1039/c0nj00278j
**26**	KERBOC	Cu^{2+}	*N*-[(pyridin-4-yl)methylidene]hydroxylamine	10.1039/D2CE01000C
**27**	KOYWEC	Rh^{3+}	biimidazole	10.1107/S2052520614021428
**28**	LAMGUC	Cu^{2+}	*N*,*N*'-bis(2-pyridylmethyl)pyrazine-2,5-dicarboxamide	10.1002/ejic.200400908
**29**	LEPZEL	Cu^{+}	formic acid	CSD communication
**30**	NIBNAO	Ag^{+}	2-(4-Pyridyl)benzimidazole	10.1016/j.poly.2006.09.102
**31**	NUJQER	Cu^{2+}	3,3'-[ethane-1,2-diylbis(dimethylsilanediyl)]dipyridine	10.1039/C9QI01581G
**32**	NUVVIK	Cu^{2+}	4-(2-Propyn-1-yl)morpholine	10.1007/s10870-009-9666-1
**33**	PEQVOX	Cu^{2+}	4-(3-Hydroxyphenyl)-1,2,4-triazole	10.1016/j.jssc.2006.05.046
**34**	QOZPIH	Cu^{2+}	pyridine-3-carboxamide	10.1134/S0036023619090055
**35**	RERVOA	Cu^{2+}	4-phenylpyridine	10.1021/cg060229b
**36**	SIJHUR	Zn^{2+}	oxydi(1,4-phenylene)bis(*N*'-(pyridin-3-yl)urea)	10.1002/asia.201800743

Table S4 continued.

**37**	TAZXAW	Cd^{2+}	m-(2,5-dioxy-3,6-dioxocyclohexa-1,4-diene-1,4-diyl)bis(dimethylsulfanium)	CSD communication
**38**	TIVSUN	Cu^{2+}	guanine	10.1039/b713960h
**39**	TOJGIJ	Ag^{+}	cis,cis,cis-1,3,5-tris(1,2,4-triazol-1-ylmethyl)-2,4,6-trimethylbenzene	10.1039/b810773d
**40**	UBOQEH	Cu^{2+}	4-amino-1,2,4-triazole	10.1039/b101936h
**41**	UKOMIU	Co^{2+}	2-(1*H*-imidazol-2-yl)-1*H*-benzimidazole-5-carboxylate	10.1002/aoc.7982
**42**	VEZPAT	Cu^{2+}	*N*-(pyridin-3-ylmethyl)benzamide	10.1039/C3CE40359A
**43**	VOXKEA	Cu^{2+}	isonicotinamide	10.1134/S0036023619090055
**44**	VUJXOO	Co^{2+}	2-(1*H*-imidazol-2-yl)-1*H*-benzimidazole-5-carboxylate	10.1002/aoc.7982
**45**	VUZNIP	Co^{2+}	3,6-bis(1*H*-imidazol-1-yl)pyridazine	CSD communication
**46**	WOXCOD	Cu^{2+}	4*H*-1,2,4-triazol-4-ylacetate	10.1002/chem.201405859
**47**	XICCUJ	Co^{2+}	1,1′-(1,2-phenylenebis(methylene))bis(1*H*-1,2,4-triazole)	10.1080/00958972.2013.769527
**48**	XIGLIK	Cd^{2+}	1,3-di(4*H*-1,2,4-triazol-4-yl)benzene	10.1039/C3CE40946E
**49**	YATSOD	Cu^{2+}	1*H*-pyrazole	10.1107/S1600536812009531
**50**	YAWRIY	Cu^{2+}	2,2'-dipyridylamine	10.1016/j.poly.2005.04.011
**51**	YOMKIW	Cu^{2+}	2-(pyridin-2-ylmethylene)hydrazinecarbothioamide	10.1016/j.poly.2014.07.032
**52**	ZOZTAL	Ag^{+}	2,2′-(5-(pyridin-4-yl)-4H-1,2,4-triazole-3,4-diyl)dipyridine	10.1016/j.jssc.2014.06.038

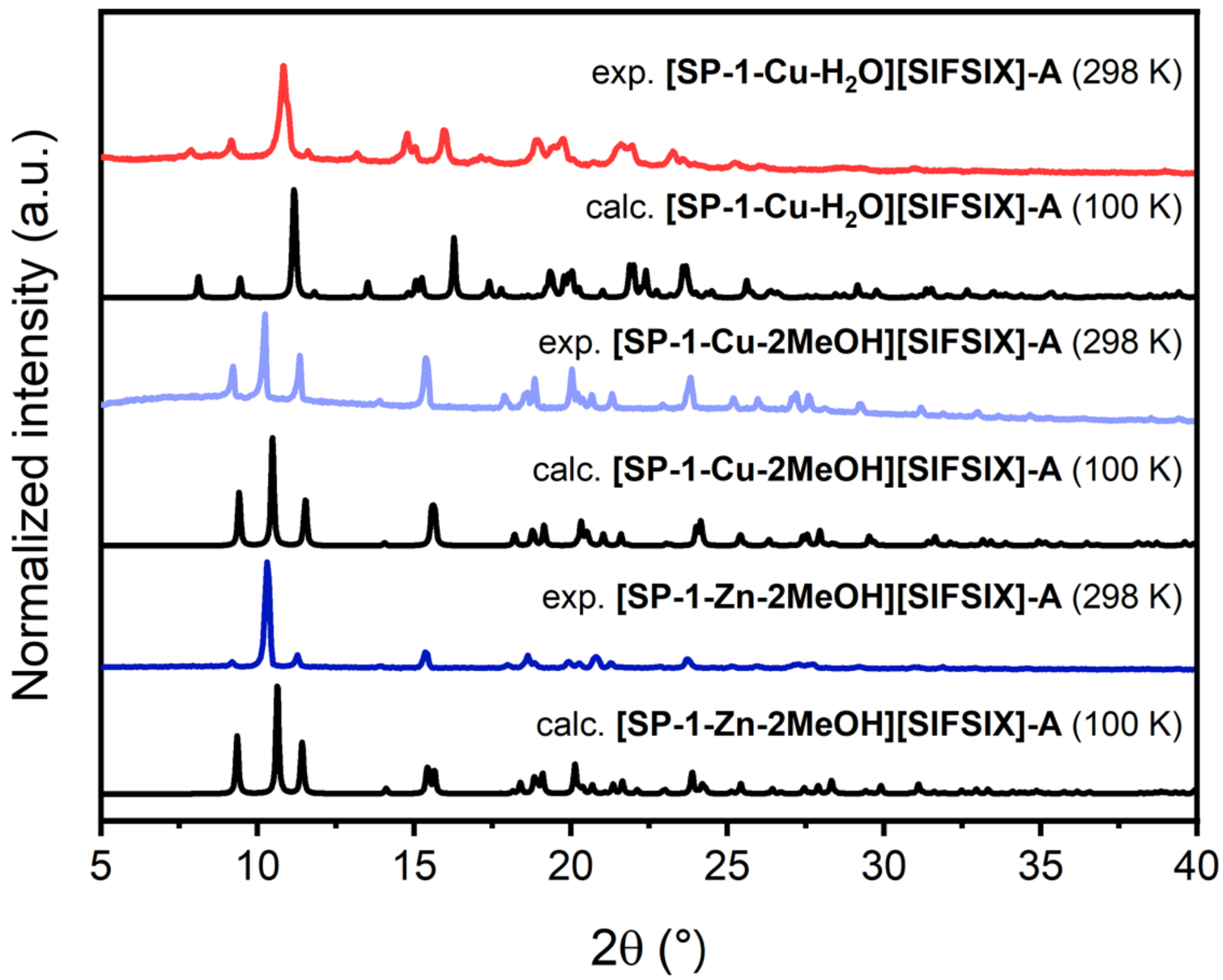

Figure S2. Calculated (black) and experimental (blue, purple, red) PXRD patterns for **[SP-1-Cu-2MeOH][SIFSIX]-A, [SP-1-Zn-2MeOH][SIFSIX]-A** and **[SP-1-Cu-H_2O][SIFSIX]-A**.

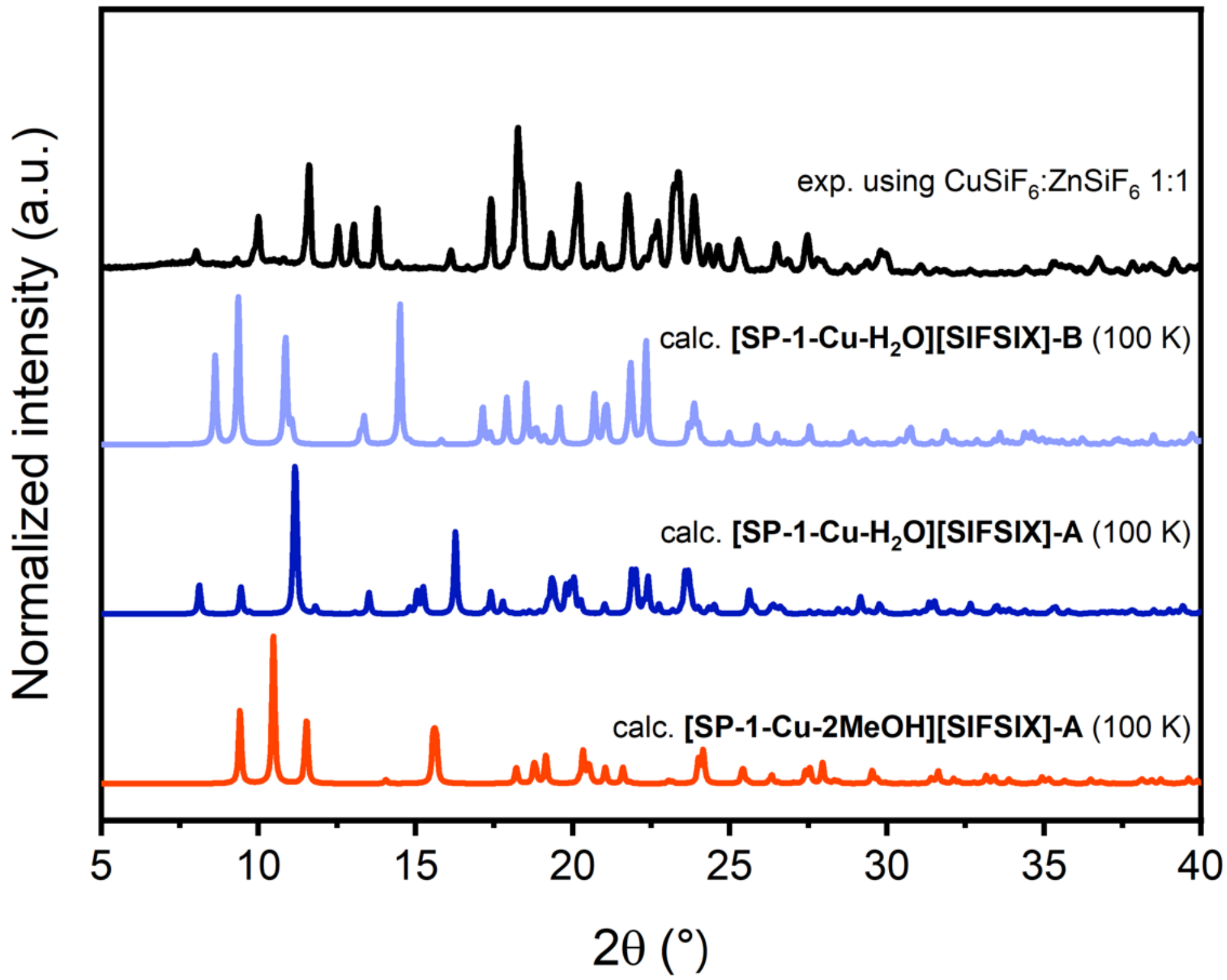

Figure S3. Calculated (orange, blue, purple) PXRD patterns for **[SP-1-Cu-2MeOH][SIFSIX]-A, [SP-1-Cu-H_2O][SIFSIX]-A**, and **[SP-1-Cu-H_2O][SIFSIX]-B**, and experimental (black) PXRD pattern for the result of an experiment using a 1:1 ratio of $CuSiF_6$ and $ZnSiF_6$.

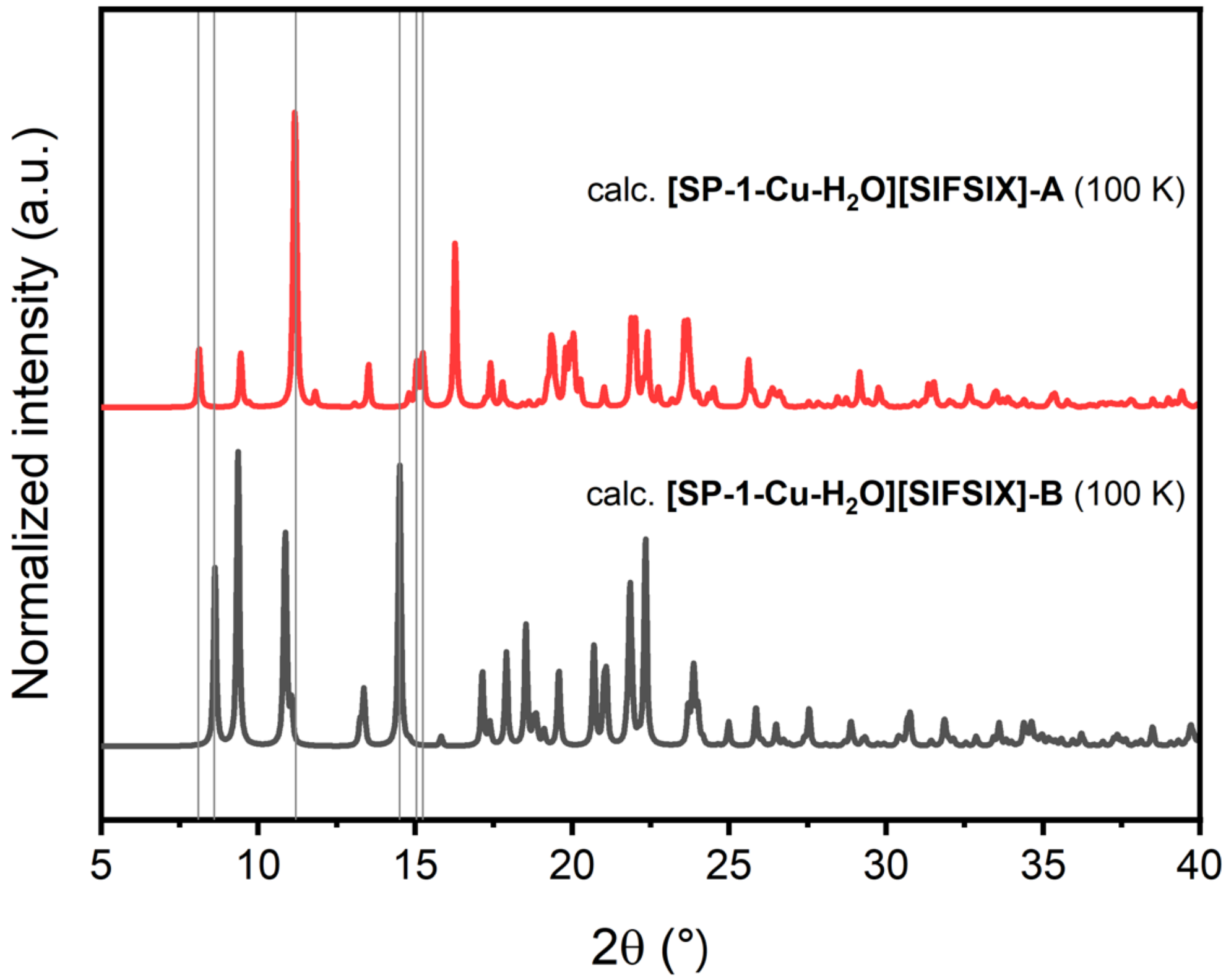

Figure S4. Comparison of PXRD patterns for **[SP-1-Cu-H_2O][SIFSIX]-A** (red) and **[SP-1-Cu-H_2O][SIFSIX]-B** (grey).

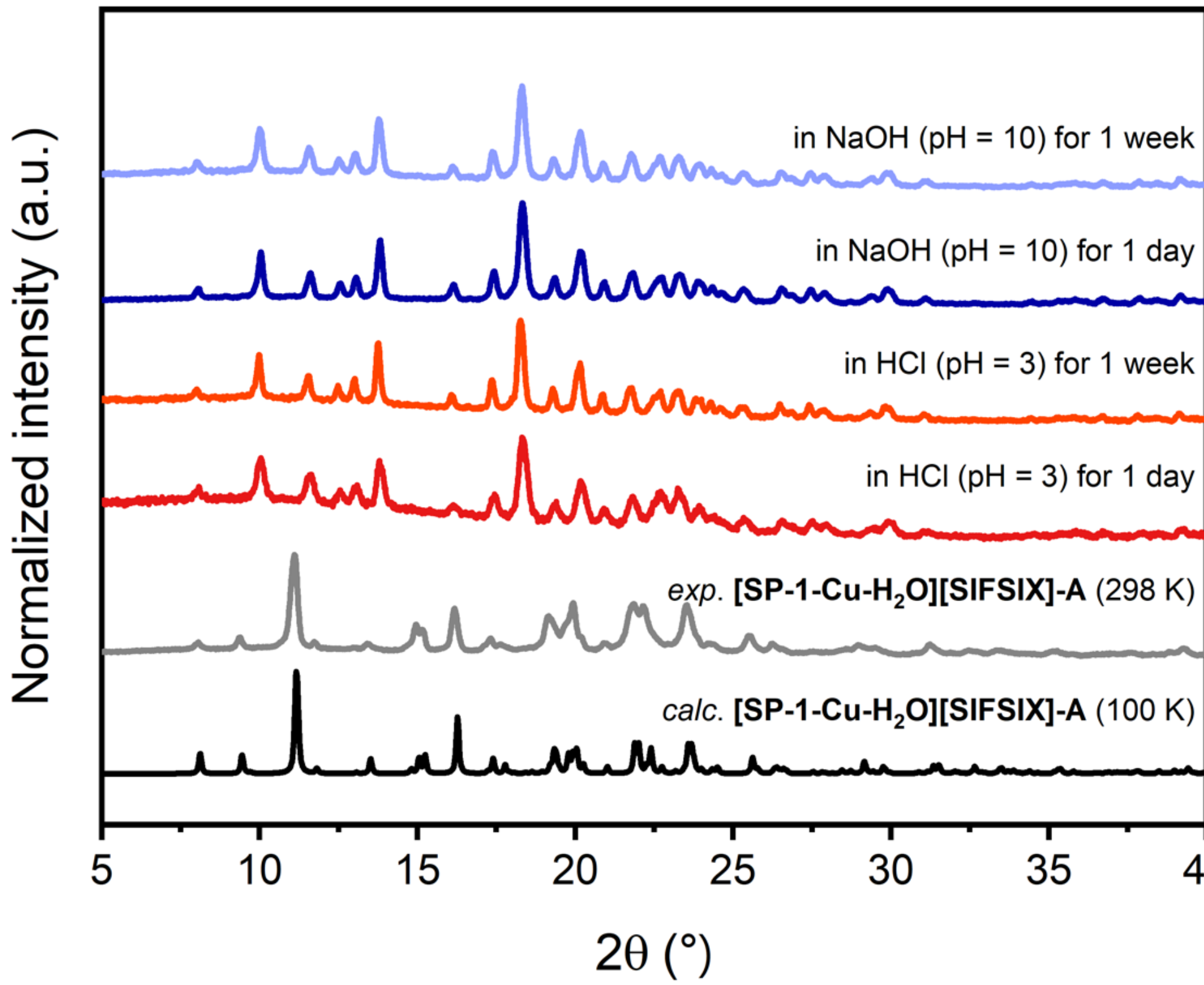

Figure S5. Calculated (black) and experimental (grey) PXRD patterns for **[SP-1-Cu-H_2O][SIFSIX]-A**, and PXRD patterns after soaking in HCl (pH = 3) for one day (red) and 1 week (orange) or in NaOH (pH = 10) for one day (blue) and 1 week (purple).

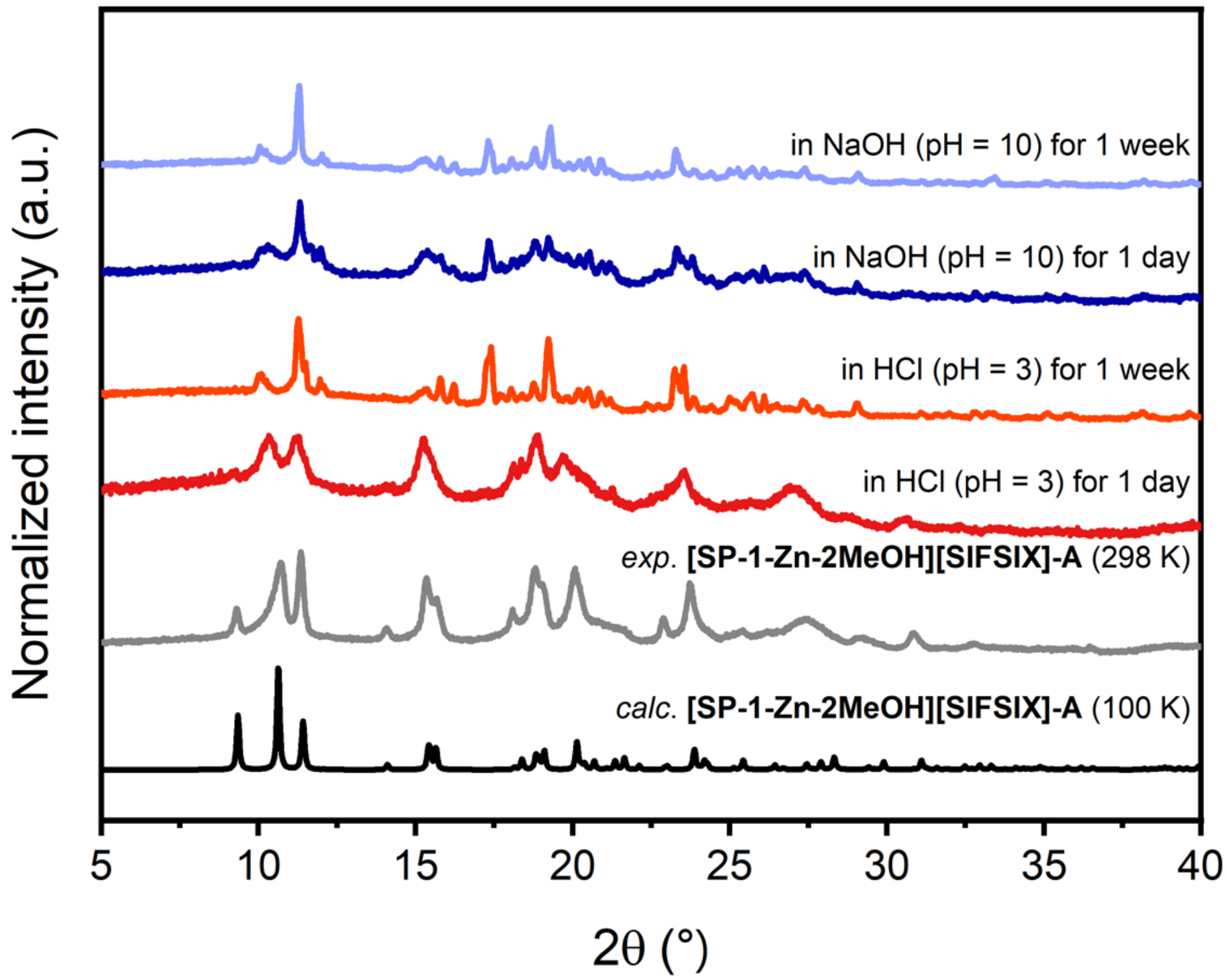

Figure S6. Calculated (black) and experimental (grey) PXRD patterns for **[SP-1-Zn-2MeOH][SIFSIX]-A**, and PXRD patterns after soaking in HCl (pH = 3) for one day (red) and 1 week (orange) or in NaOH (pH = 10) for one day (blue) and 1 week (purple).

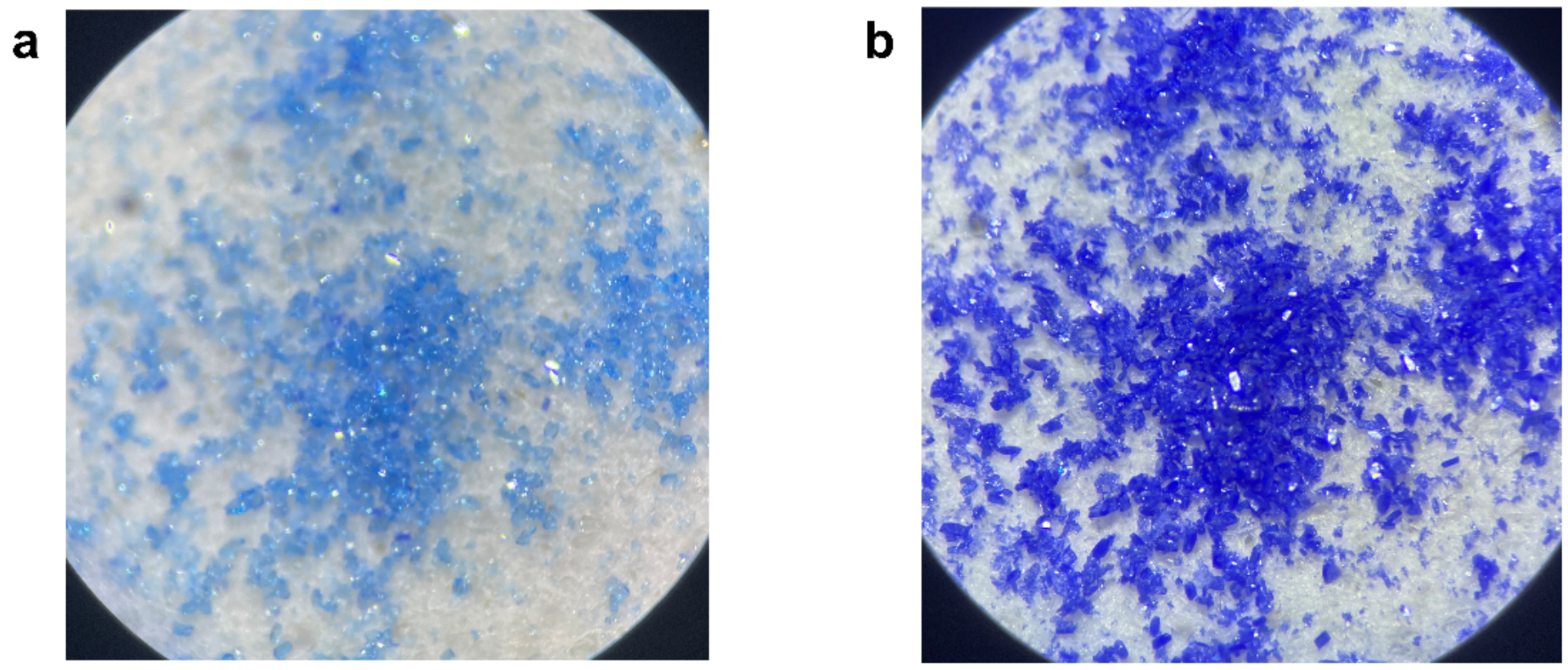

Figure S7. Color change from (a) **[SP-1-Cu-2MeOH][SIFSIX]-A** to (b) **[SP-1-Cu-H_2O][SIFSIX]-A** upon drying in air for 10 minutes.

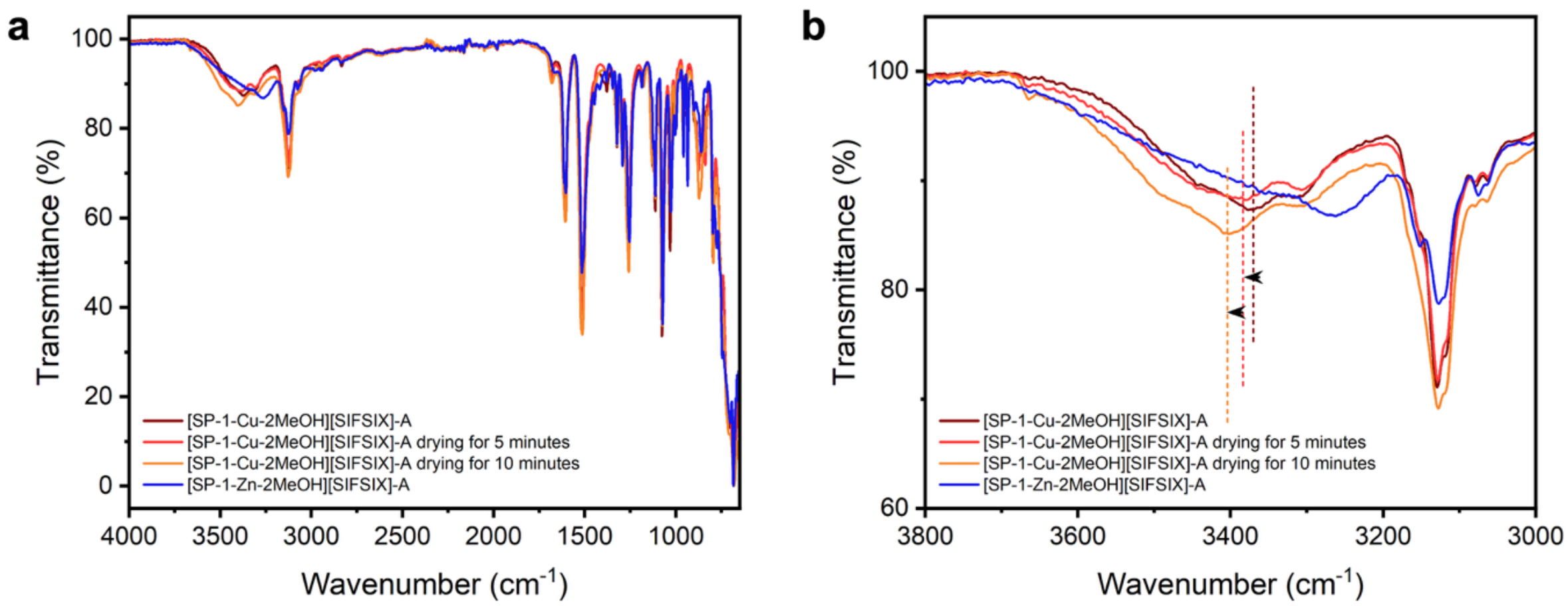

Figure S8. (a) FTIR spectra for **[SP-1-Cu-2MeOH][SIFSIX]-A** (wine red), **[SP-1-Cu-2MeOH][SIFSIX]-A** drying in air for 5 (red) and 10 (orange) minutes, and **[SP-1-Zn-2MeOH][SIFSIX]-A** (blue). (b) Inlet showing the bands in the region of 3800–3000 cm^{-1}.

Table S5. Angles around the coordination sphere of Cu^{2+} in **[SP-1-Cu-H_2O][SIFSIX]-A** and **[SP-1-Cu-H_2O][SIFSIX]-B**.

Compound	∠N–Cu–N (˚)	∠N–Cu–O (˚)
**[SP-1-Cu-H_2O][SIFSIX]-A**	87.0(3)	97.6(3)
	86.3(3)	97.4(3)
	93.3(3)	93.3(3)
	91.4(3)	93.6(3)
**[SP-1-Cu-H_2O][SIFSIX]-B**	89.6(3)	95.8(3)
	87.4(3)	105.0(3)
	83.3(3)	96.0(3)
	91.8(3)	91.8(3)

Table S6. Calculations for the density of the pseudopolymorphs **[SP-1-Cu-H_2O][SIFSIX]-A** and **[SP-1-Cu-H_2O][SIFSIX]-B**, and **[SP-1-Cu-2MeOH][SIFSIX]-A**. The formula weight is calculated without the solvents.

Compound	Formula weight (g/mol)	Z	Unit cell volume (Å^3)	Density (g/cm^3)
**[SP-1-Cu-H_2O][SIFSIX]-A**	644.1	4	2754	1.553
**[SP-1-Cu-H_2O][SIFSIX]-B**	644.1	16	11417	1.499
**[SP-1-Cu-2MeOH][SIFSIX]-A**	345.1	8	2967	1.545

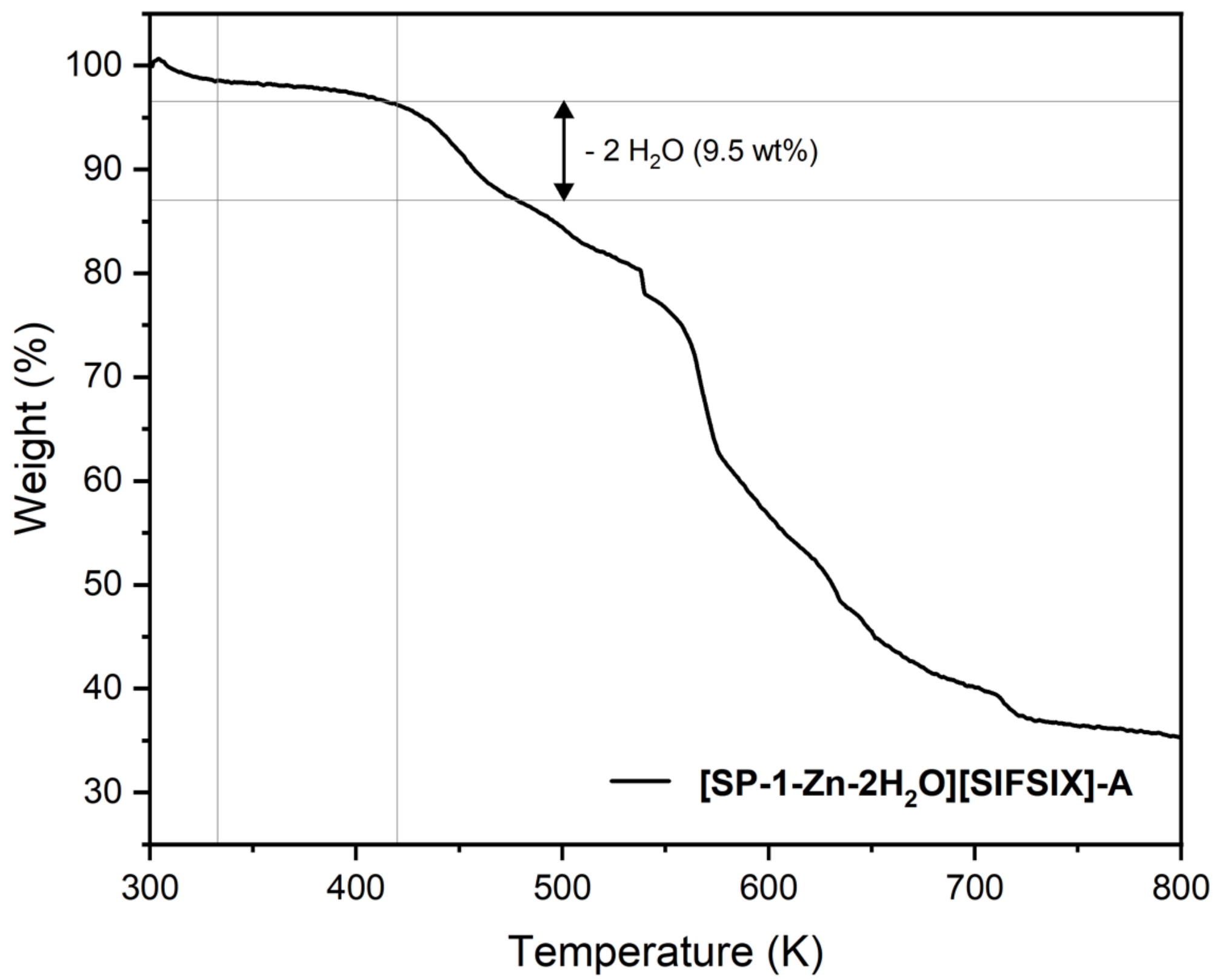

Figure S9. Thermogravimetric trace for **[SP-1-Zn-2H_2O][SIFSIX]-A** under nitrogen flow.

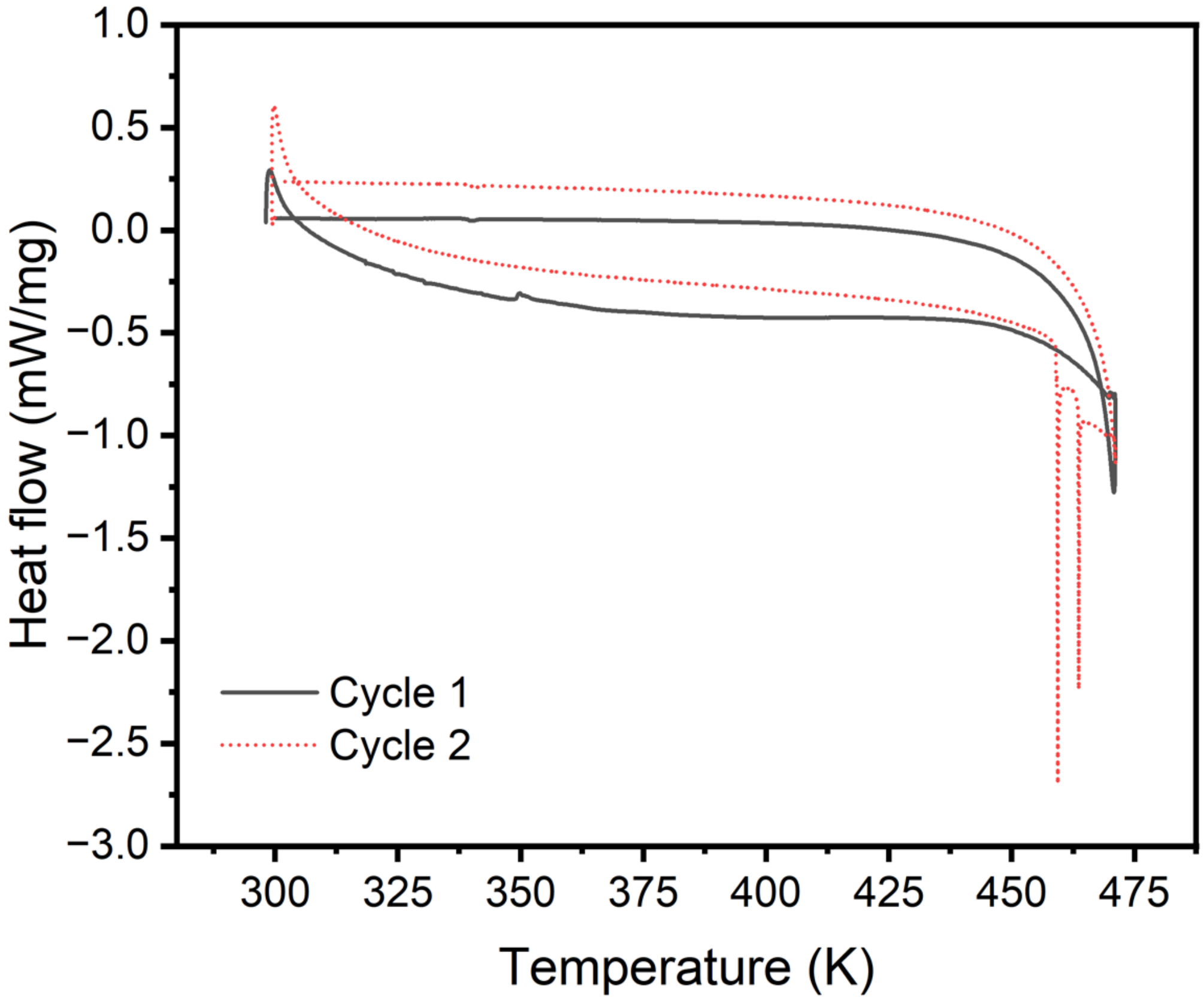

Figure S10. Two cycles of differential scanning calorimetry for **[SP-1-Zn-2H_2O][SIFSIX]-A** under nitrogen flow.

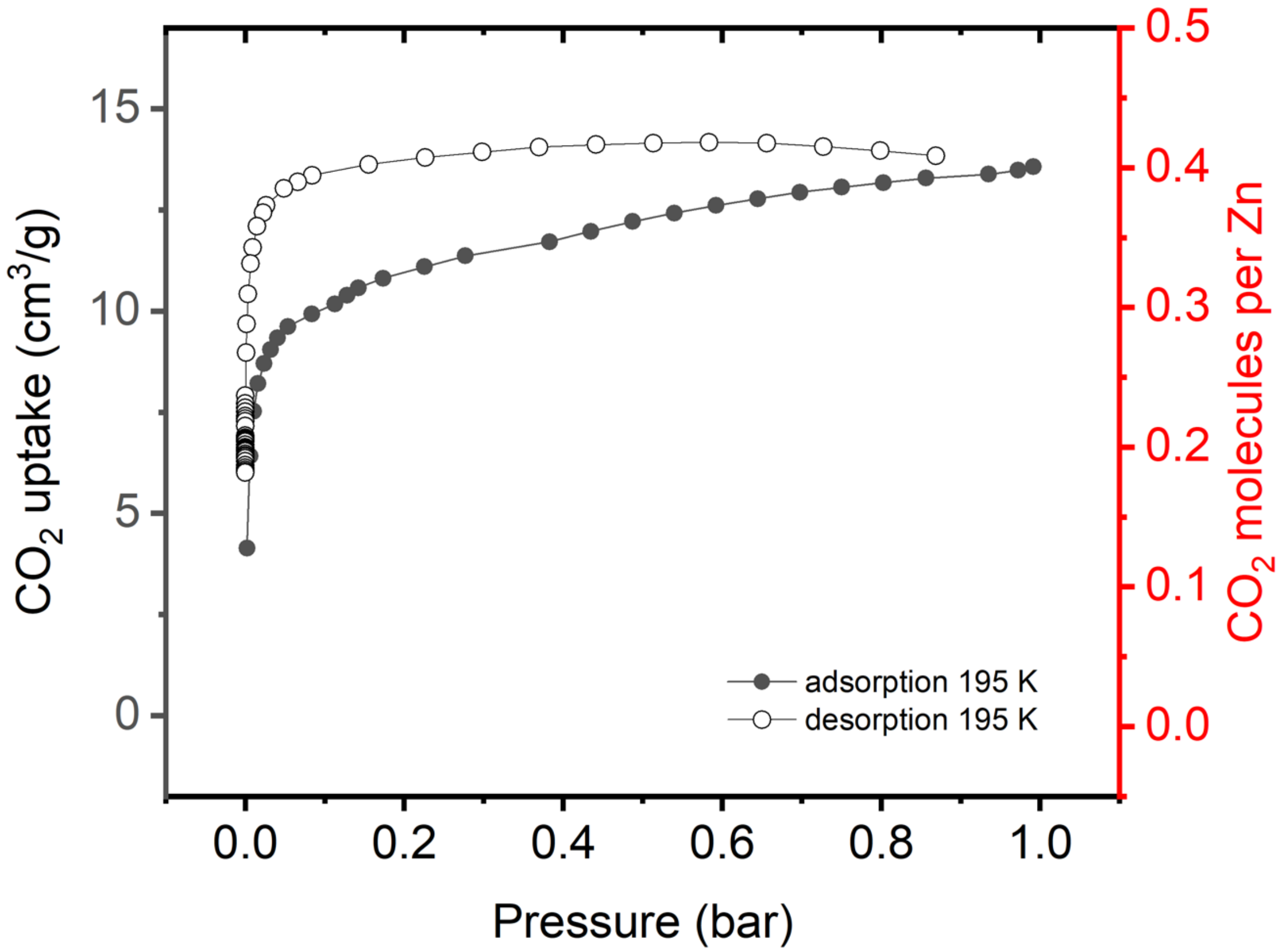

Figure S11. CO_2 sorption isotherm for **[SP-1-Zn-2H$_2$O][SIFSIX]-A** collected at 195 K. Full circles = adsorption; open circles = desorption.

Table S7. Calculation of CO_2 uptake per Zn at 298 K and 1 bar.

Formula	Molar mass	CO_2 uptake (cm^3/g)	CO_2 uptake (molecules per FU)	CO_2 uptake (molecules per Zn)
$C_{12}H_{12}N_4OZn_{0.5}$, $F_3Si_{0.5}$	331.99	14.7	0.22	0.44

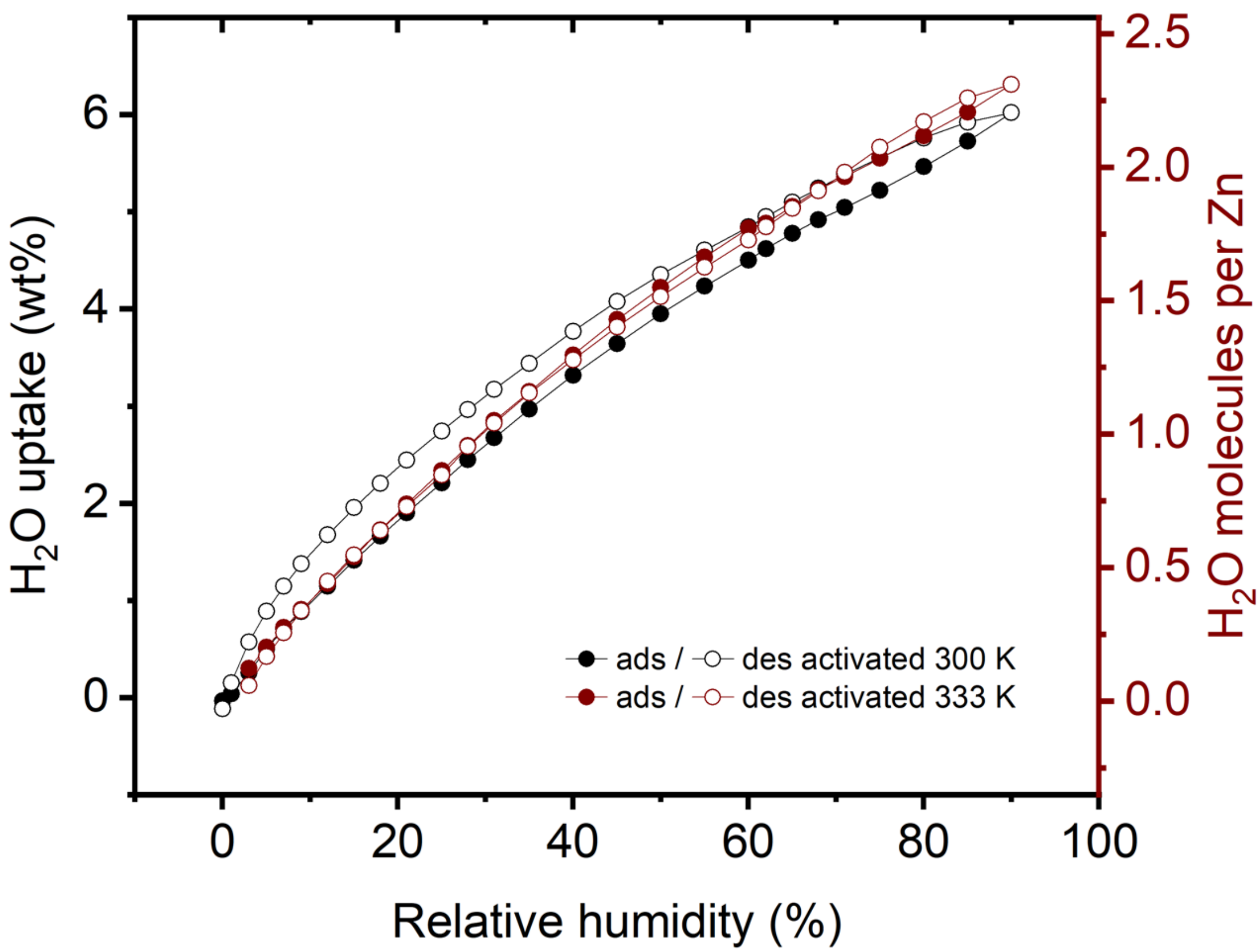

Figure S12. Dynamic vapour sorption (DVS) of water vapour for **[SP-1-Zn-2H_2O][SIFSIX]-A**, upon activation at 300 K (black) and 333 K (wine red). Full circles = adsorption; open circles = desorption.

Table S8. Calculation of H_2O uptake per Zn at 300 K and 90% RH.

Formula	Molar mass	H_2O uptake (wt%)	H_2O uptake (molecules per FU)	H_2O uptake (molecules per Zn)
$C_{12}H_{12}N_4OZn_{0.5}$, $F_3Si_{0.5}$	331.99	5.97	1.10	2.20

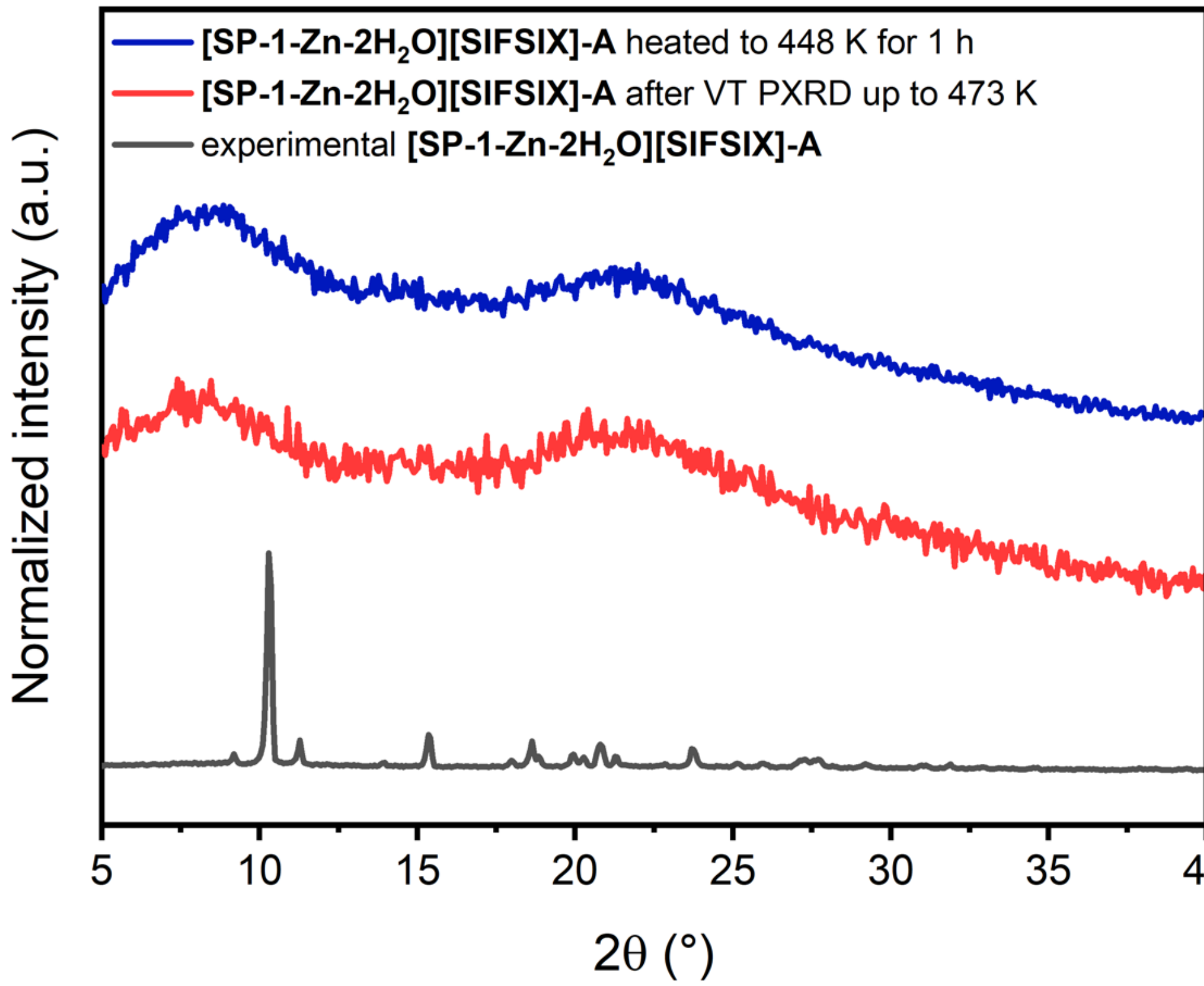

Figure S13. Calculated (black) and experimental (red, blue) PXRD patterns for **[SP-1-Zn-2H_2O][SIFSIX]-A** upon heating to 473 K (red) and 448 K (blue).

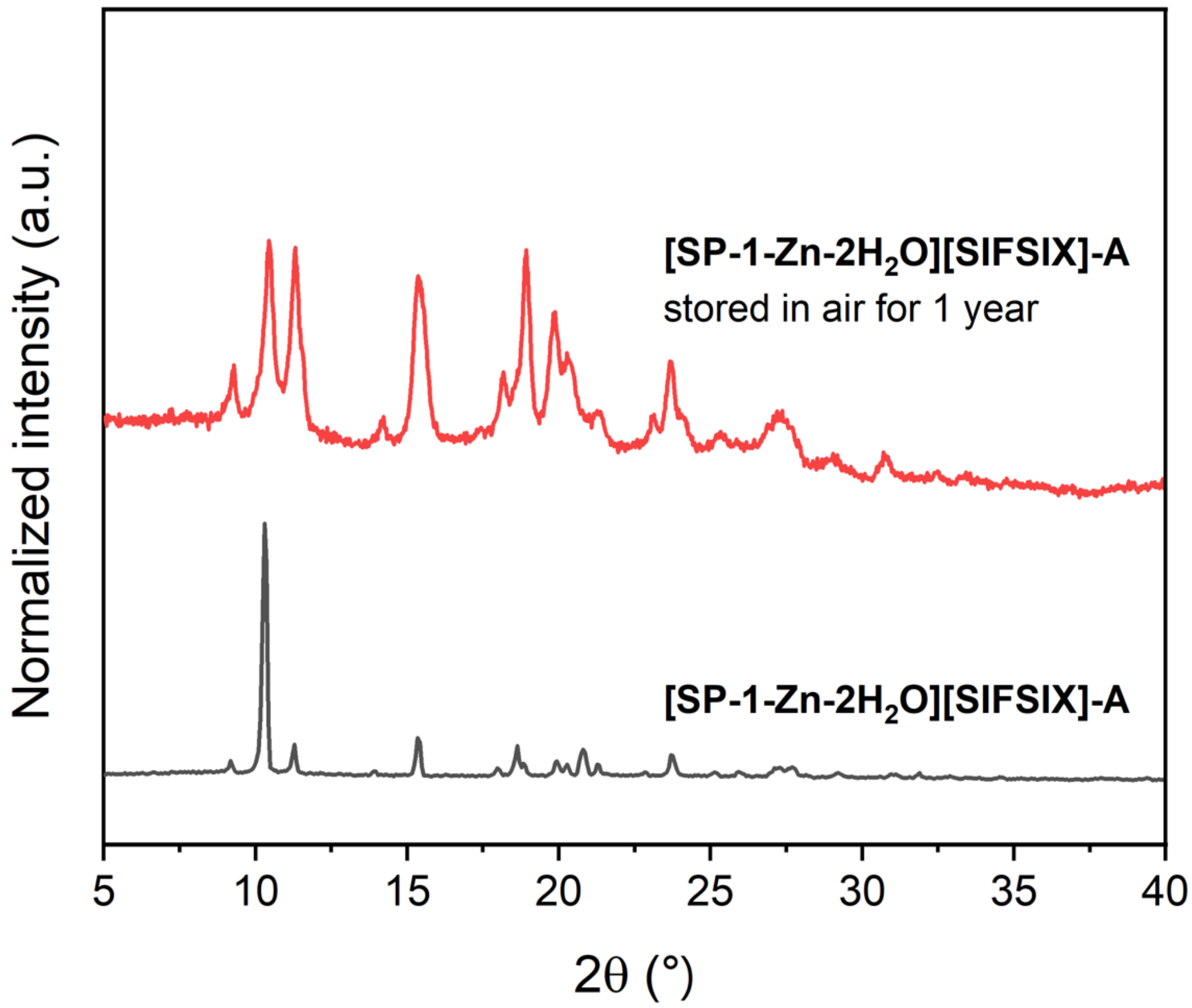

Figure S14. Experimental PXRD patterns for **[SP-1-Zn-2H_2O][SIFSIX]-A** upon initial synthesis (black) and upon being kept in air for 1 year (red).

References

1. R. H. Lam, S. T. Keaveney, B. A. Messerle and I. Pernik, ACS Catal., 2023, 13, 1999–2010.
2. A. S. Degtyarenko, E. B. Rusanov, A. Bauzá, A. Frontera, H. Krautscheid, A. N. Chernega, A. A. Mokhir and K. V. Domasevitch, Chem. Commun., 2013, 49, 9018–9020.
3. R. Hoshino and S. Adachi, J. Appl. Phys., 2013, 114.
4. C. R. Groom, I. J. Bruno, M. P. Lightfoot and S. C. Ward, Struct. Sci., 2016, 72, 171–179.
5. B. D. S. Visualizer, San Diego, USA, 2020.
